# The β-1,3-glucanosyltransferase Gas1 regulates Sir2-mediated rDNA stability in *Saccharomyces cerevisiae*

**DOI:** 10.1093/nar/gku570

**Published:** 2014-06-30

**Authors:** Cheol Woong Ha, Kwantae Kim, Yeon Ji Chang, Bongkeun Kim, Won-Ki Huh

**Affiliations:** Department of Biological Sciences and Research Center for Functional Cellulomics, Institute of Microbiology, Seoul National University, Seoul 151-747, Republic of Korea

## Abstract

In *Saccharomyces cerevisiae*, the stability of highly repetitive rDNA array is maintained through transcriptional silencing. Recently, a β-1,3-glucanosyltransferase Gas1 has been shown to play a significant role in the regulation of transcriptional silencing in *S. cerevisiae*. Here, we show that the *gas1*Δ mutation increases rDNA silencing in a Sir2-dependent manner. Remarkably, the *gas1*Δ mutation induces nuclear localization of Msn2/4 and stimulates the expression of *PNC1*, a gene encoding a nicotinamidase that functions as a Sir2 activator. The lack of enzymatic activity of Gas1 or treatment with a cell wall-damaging agent, Congo red, exhibits effects similar to those of the *gas1*Δ mutation. Furthermore, the loss of Gas1 or Congo red treatment lowers the cAMP-dependent protein kinase (PKA) activity in a cell wall integrity MAP kinase Slt2-dependent manner. Collectively, our results suggest that the dysfunction of Gas1 plays a positive role in the maintenance of rDNA integrity by decreasing PKA activity and inducing the accumulation of Msn2/4 in the nucleus. It seems that nuclear-localized Msn2/4 stimulate the expression of Pnc1, thereby enhancing the association of Sir2 with rDNA and promoting rDNA stability.

## INTRODUCTION

In *Saccharomyces cerevisiae*, 100–200 copies of a 9*.*1-kb ribosomal DNA (rDNA) repeat exist as a tandem array on chromosome XII (1). Each rDNA repeat contains the RNA polymerase (Pol) I-transcribed 35S rRNA gene and the non-transcribed spacer (NTS) region, which is divided by the Pol III-transcribed 5S rRNA gene into NTS1 and NTS2. Because of its highly repetitive nature, the rDNA array is intrinsically unstable and an easy target for homologous recombination. Homologous recombination between rDNA repeats leads to the formation of extrachromosomal rDNA circles, which accumulate to toxic levels in mother cells and cause aging in *S. cerevisiae* (2). Under normal conditions, rDNA repeats remain relatively stable because recombination between rDNA repeats is negatively regulated through a mechanism referred to as rDNA silencing.

Sir2 is an NAD^+^-dependent histone deacetylase (3–5) and a subunit of the rDNA silencing complex called RENT (regulator of nucleolar silencing and telophase exit), which represses Pol II-dependent transcription at the rDNA locus (6). Sir2 plays a crucial role in stabilizing rDNA repeats, thereby extending replicative lifespan (2,7,8). The enzymatic activity of Sir2 is regulated by an endogenous level of nicotinamide, a physiological inhibitor of Sir2 (9). The *PNC1* gene encodes a nicotinamidase that converts nicotinamide to nicotinic acid as part of the NAD^+^ salvage pathway (10). Therefore, the deletion of *PNC1* results in an elevated level of nicotinamide in cells that can inhibit Sir2 activity. Nicotinamide also prevents the association of Sir2 with rDNA (11). The expression of Pnc1 is stimulated during the stationary phase of growth, by various hyperosmotic shocks or by ethanol treatment. (10). The *PNC1* promoter contains a binding site for the stress-responsive transcription factors, Msn2 and Msn4. Msn2/4 actually regulate Pnc1 expression and Sir2-mediated longevity (12).

Msn2/4 are normally maintained in the cytoplasm. However, stress or glucose depletion induces rapid nuclear accumulation of Msn2/4 (13,14). This localization change is negatively controlled by the cAMP-PKA pathway through Msn2/4 phosphorylation. As cAMP levels fall, the cAMP-dependent protein kinase (PKA) activity is downregulated, and dephosphorylation of the Msn2 nuclear localization signal promotes nuclear import of Msn2 (13,15,16). Nuclear export of Msn2 is dependent on the nuclear exportin Msn5 (15). It is also known that Msn2/4 are activated by protein kinases Mck1, Rim15, Yak1, Snf1 and Hog1, and phosphatases Psr1, Psr2 and Glc7 (17).

Gas1 is β-1,3-glucanosyltransferase and plays a role in the formation and maintenance of β-1,3-glucan chains and cell wall assembly (18). Gas1 elongates and rearranges β-1,3-glucan side chains, which are crosslinked with β-1,6-glucan, chitin and proteins to form the main layer of the cell wall (19,20). Consistent with the role of Gas1 in cell wall biogenesis, *gas1*Δ cells exhibit weakened cell wall phenotypes, such as abnormal cell wall morphology and sensitivity to cell wall-perturbing drugs. Recently, Koch and Pillus (21) reported a surprising role for Gas1 in the regulation of transcriptional silencing in *S. cerevisiae*; they showed that telomeric silencing is reduced and rDNA silencing is enhanced in *gas1*Δ cells. They found that the silencing effects exhibited in *gas1*Δ cells were caused by the loss of the enzymatic activity of Gas1 and that Gas1 physically interacted with Sir2; thus, they suggested that Gas1 may add a carbohydrate moiety to Sir2 or other factors that interact with Sir2 and thereby alter the enzymatic activity of Sir2. However, whether Sir2 is actually modified by Gas1 or, if not, how the loss of the enzymatic activity of Gas1 affects transcriptional silencing remains unknown.

In the present study, we sought to further investigate the role of Gas1 in transcriptional silencing of rDNA in *S. cerevisiae*. We observed that the deletion of *GAS1* induced the translocalization of the stress-responsive transcription factors Msn2/4 from the cytoplasm to the nucleus. In *gas1*Δ cells, the binding of Msn2/4 to the *PNC1* promoter was increased and the expression of *PNC1* was stimulated. Consistent with previous reports (11,12), *gas1*Δ cells with increased Pnc1 expression exhibited enhanced association of Sir2 with rDNA and increased rDNA stability. We also found that the loss of Gas1 lowered PKA activity in a cell wall integrity MAP kinase Slt2-dependent manner. Taken together, our results suggest that the dysfunction of Gas1 plays a positive role in the maintenance of rDNA integrity by decreasing PKA activity and inducing the accumulation of Msn2/4 in the nucleus. It seems that nuclear-localized Msn2/4 stimulate the expression of Pnc1, which then leads to enhancing the association of Sir2 with rDNA and promoting rDNA stability.

## MATERIALS AND METHODS

### Yeast strains, plasmids and growth conditions

Yeast strains used in this study are listed in Supplementary Table S1. Yeast strains were genetically manipulated according to the one-step PCR-mediated gene targeting procedure, as previously described (22). For the construction of the Gas1-expressing plasmids, DNA fragments containing *GAS1-TAP* or *GAS1-GFP* sequences were amplified by PCR and cloned into pRS413 or pRS415 vectors. The enzymatically inactive Gas1 mutant, Gas1^E161Q, E262Q^, was obtained by the QuickChange multisite-directed mutagenesis protocol (Stratagene). All constructs were verified by DNA sequencing. pRS423-pr*^CUP^-6×MYC-cki1^2^*^−*200(S125*^*^/130A)^* has been described previously (23,24). Rich medium (YPD; 1% yeast extract, 2% peptone, 2% glucose) and synthetic complete (SC) medium lacking appropriate amino acids for selection were prepared as previously described (25). Unless otherwise noted, cells were grown to an OD_600_ of 1.0 in YPD medium at 30°C.

### rDNA silencing assay

Silencing at the rDNA region was assayed as previously described (26,27). Yeast cells were grown to an OD_600_ of 1.0, and 3 μl of 10-fold serial dilutions of the cell suspensions was spotted on the appropriate media. Plates were incubated at 30°C for 3 days.

### Quantification of *mURA3* and *PNC1* mRNA

Total RNA was isolated from yeast cells using the RNeasy MiniKit (Qiagen). cDNA for reverse transcription-PCR was generated using the ProtoScript First Strand cDNA Synthesis Kit (New England Biolabs). The *mURA3* silencing reporter gene, which contains the *TRP1* promoter instead of the *URA3* promoter, has been described previously (28). The amount of *mURA3, PNC1* and *ACT1* mRNA was analyzed by quantitative real-time reverse transcription-PCR using the Applied Biosystems 7300 Real-Time PCR system. For the calculation of relative *mURA3* transcript level, the transcript levels of the reporter *mURA3* gene inside (*RDN1-NTS1*::*mURA3*) or outside the rDNA locus (*leu2*::*mURA3*) in a given strain were measured and normalized against *ACT1* mRNA level. Then the ratio of normalized *mURA3* transcript level at NTS1 to that at *leu2* was calculated. For the calculation of relative *PNC1* transcript level, the transcript level of the *PNC1* gene in a given strain was measured and normalized against that of *ACT1*, and fold increases were calculated using the 2^−ΔΔC^_T_ method (29). The primers used for the amplification of *mURA3* were 5′-CTGTTGACATTGCGAAGAGC-3′ and 5′-TCTCCCTTGTCATCTAAACC-3′, those used for the amplification of *PNC1* were 5′-AGACGAGGTTTACATTGTCG-3′ and 5′-CCTTCAACTCTTCCTTAACC-3′, and those used for the amplification of *ACT1* were 5′-TGACTGACTACTTGATGAAG-3′ and 5′-TGCATTTCTTGTTCGAAGTC-3′. All reactions were carried out in three independent experiments. Statistical analysis was performed using a two-tailed Student's *t*-test.

### rDNA recombination assay

The rDNA recombination rate was determined by measuring the frequency of the loss of *ADE2* integrated at the rDNA locus of strain DMY3010 as previously described (7). Exponentially growing cells (OD_600_ ∼ 1.0) in SC medium were sonicated briefly to prevent aggregation and were spread on SC plates. Colonies were allowed to grow for 3 days at 30°C and then placed at 4°C for 3 days to enhance color development. Colonies in which the *ADE2* marker had been lost accumulated a red pigment, while colonies that maintained and expressed *ADE2*, which is weakly silenced in the rDNA region, remained white. A marker loss event during the first division after plating resulted in half-red/half-white colonies. The rDNA recombination rate was calculated by dividing the number of half-red/half-white colonies by the total number of colonies. Entirely red colonies were excluded from all calculations. Three independent experiments were performed, and more than 20 000 colonies were examined for each assay. Statistical analysis was performed using a two-tailed Student's *t*-test.

### Fluorescence microscopy

Fluorescence microscopy was performed on a Nikon Eclipse Ti inverted microscope. Cells were grown in SC medium at 30°C. Image analysis was performed using the NIS-Elements AR3.1 microscopy software (Nikon) in order to determine the percentage of cells with predominately nuclear fluorescence. At least 100 cells were counted for each determination.

### Chromatin immunoprecipitation (ChIP) assay

ChIP assays were performed as previously described (11). For TAP ChIP experiments, pre-washed IgG Sepharose beads (17-0969-01, GE Healthcare) were used. ChIP samples were analyzed by quantitative real-time PCR using SYBR Green and the Applied Biosystems 7300 real-time PCR system. The sequences of PCR primers used in ChIP experiments are shown in Supplementary Table S2. Relative fold enrichment of Msn2/4 at the endogenous *PNC1* promoter region was analyzed by calculating the ratio of the *PNC1* promoter to *ACT1*, an internal control, as follows: [*PNC1*(IP)/*ACT1*(IP)]/[*PNC1*(input)/*ACT1*(input)]. Relative fold enrichment of Sir2 at the rDNA region was determined by calculating the ratio of rDNA to *CUP1*, an internal control, as follows: [rDNA(IP)/*CUP1*(IP)]/[rDNA(input)/*CUP1*(input)]. Each set of experiments was performed at least three times. Statistical analysis was performed using a two-tailed Student's *t*-test.

### Western blot analysis

Whole-cell extracts were run on sodium dodecyl sulfate (SDS)-polyacrylamide gel electrophoresis. Western blot analysis was performed by standard methods using a mouse anti-GFP antibody (sc-9996, Santa Cruz Biotechnology) for the detection of GFP-tagged proteins, an HRP-conjugated anti-mouse IgG antibody (A9044, Sigma) for the detection of TAP-tagged proteins, an HRP-conjugated anti-HA antibody (sc-7392, Santa Cruz Biotechnology) for the detection of HA-tagged proteins, and a mouse anti-Myc antibody (sc-40, Santa Cruz Biotechnology) for the detection of Myc-tagged proteins. Actin and hexokinase were used as loading controls and were detected by an anti-actin antibody (sc-1616, Santa Cruz Biotechnology) and an anti-hexokinase antibody (H2035-02, United States Biological), respectively. Each western blotting experiment was performed at least three independent times. Images were captured using a luminescent image analyzer LAS-3000 (Fujifilm) and quantification of captured images was performed using Multi Gauge V3.0 software (Fujifilm).

### Intracellular NAD^+^ measurement

Intracellular NAD^+^ measurement was performed as previously described with some modification (30). Yeast cells were grown in 50 ml YPD medium to OD_600_ ∼ 1.4 and then harvested by centrifugation. Cell pellets were extracted for 30 min with 500 μl of ice-cold 1 M formic acid (saturated with butanol). 125 μl of ice-cold 100% trichloroacetic acid was added and incubated on ice for 15 min. The mixture was centrifuged at 4000 × *g* for 20 min at 4°C and the acid-soluble supernatant was saved. The pellet was re-extracted with 250 μl of 20% trichloroacetic acid and pelleted again. The supernatants were combined and used for NAD^+^ measurement. 150 μl of the acid extract was added to 850 μl of reaction buffer containing 300 mM Tris–HCl (pH 9.7), 200 mM lysine HCl, 0.2% ethanol and 150 μg/ml alcohol dehydrogenase (A7011, Sigma). Reactions were incubated at 30°C for 20 min, and absorbance was measured at 340 nm. A base-line correction was made by subtracting the absorbance of a reaction without alcohol dehydrogenase. The intracellular NAD^+^ concentration was calculated from the extinction coefficient as previously described (31). Each set of experiments was performed at least three independent times. Statistical analysis was performed using a two-tailed Student's *t*-test.

## RESULTS

### Loss of Gas1 increases rDNA silencing and promotes rDNA stability in the presence of Sir2

To investigate the role of Gas1 in transcriptional silencing of rDNA, we performed a spot assay using yeast strains carrying the *mURA3* silencing reporter gene inserted either inside the NTS1 region of the rDNA locus (*RDN1-NTS1*::*mURA3*) or outside the rDNA array (*leu2*::*mURA3*) (26). Cells were spotted in 10-fold serial dilutions on SC medium as a plating control and on SC medium without uracil to monitor silencing of the *mURA3* reporter gene. The *mURA3* reporter gene was efficiently silenced at the rDNA region in wild-type cells, while silencing of *mURA3* at the rDNA region was abolished in *sir2*Δ cells (Supplementary Figure S1A). Consistent with a previous report (21), *gas1*Δ cells exhibited enhanced silencing of *mURA3* at the rDNA region compared with wild-type cells. *gas1*Δ *sir2*Δ cells showed a defect in rDNA silencing similar to that of *sir2*Δ cells.

To confirm the above spot assay results, we performed real-time reverse transcription-PCR to measure the transcript levels of the Pol II-transcribed *mURA3* gene integrated inside (*RDN1-NTS1*::*mURA3*) or outside the rDNA array (*leu2*::*mURA3*). In wild-type cells, the reporter *mURA3* gene was efficiently silenced at the rDNA locus (∼50%) compared with outside the rDNA locus (Supplementary Figure S1B). As expected, silencing of the reporter gene at the rDNA locus was further enhanced in *gas1*Δ cells. In *sir2*Δ and *gas1*Δ *sir2*Δ cells, the transcript levels of *mURA3* inside the rDNA locus were similar to those of *mURA3* outside the rDNA locus, suggesting that silencing of *mURA3* at the rDNA locus was almost completely abolished in these cells. These results indicate that the loss of Gas1 increases rDNA silencing and that the effect of Gas1 on rDNA silencing is dependent on the presence of Sir2.

We next investigated whether the loss of Gas1 could promote rDNA stability by reducing rDNA recombination. To measure the rDNA recombination rate, the frequency of the loss of the *ADE2* marker gene integrated at the rDNA locus was monitored (7). In accordance with the above observation that the loss of Gas1 increases rDNA silencing, *gas1*Δ cells showed a significant decrease in the rate of *ADE2* marker loss compared with wild-type cells (Supplementary Figure S1C). This result suggests that the absence of Gas1 contributes to the repression of rDNA recombination and to the promotion of rDNA stability. As shown previously (26,32), the deletion of *SIR2* considerably increased the rate of marker loss. The rDNA recombination was similarly increased in *gas1*Δ *sir2*Δ cells. Taken together, these observations demonstrate that the loss of Gas1 increases rDNA silencing and promotes rDNA stability in a Sir2-dependent manner.

### Loss of Gas1 promotes Sir2-mediated rDNA silencing by inducing the expression of Pnc1 in an Msn2/Msn4-dependent manner

**Figure 1.**
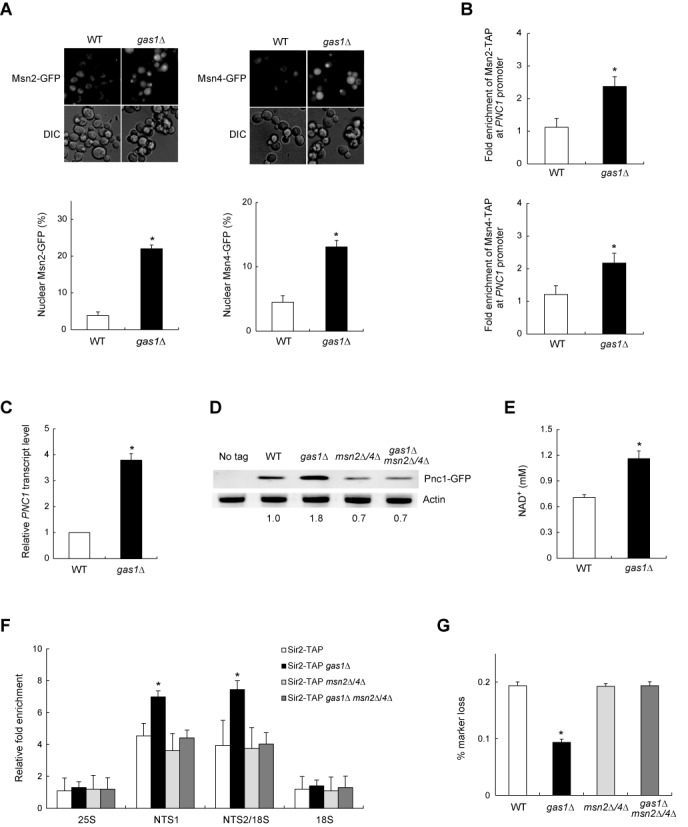
The loss of Gas1 induces the expression of Pnc1 and promotes rDNA stability in an Msn2/4-dependent manner. (**A**) The absence of Gas1 promotes nuclear accumulation of Msn2 and Msn4. Cells with chromosomally GFP-tagged Msn2 or Msn4 were grown to logarithmic phase in SC medium and analyzed by fluorescence microscopy (upper panels). Shown below is the percentage of nuclear Msn2 (lower left panel) and Msn4 (lower right panel) in wild-type (WT) and *gas1*Δ cells. Values represent the average of three independent experiments and at least 100 cells were counted for each determination. Error bars indicate the standard deviation. Asterisks indicate *P* < 0.05, compared with WT cells (two-tailed Student's *t*-test). (**B**) The association of Msn2/4 with the *PNC1* promoter region is enhanced in the absence of Gas1. The degree of association of Msn2 (upper panel) and Msn4 (lower panel) with the *PNC1* promoter region was measured using a ChIP assay in WT and *gas1*Δ cells. Values represent the average of three independent experiments, and error bars indicate the standard deviation. Asterisks indicate *P* < 0.05, compared with WT cells (two-tailed Student's *t*-test). (**C**) The loss of Gas1 promotes the *PNC1* transcript level. Total RNA was extracted from WT and *gas1*Δ cells. Quantitative real-time reverse transcription-PCR analysis was performed to measure the transcript level of *PNC1*. Amplification efficiencies were validated and normalized against *ACT1*. The relative *PNC1* transcript level was calculated as the ratio of the normalized transcript level of the *PNC1* to that of *ACT1*. Values represent the average of three independent experiments, and error bars indicate the standard deviation. Asterisks indicate *P* < 0.05, compared with WT cells (two-tailed Student's *t*-test). (**D**) The protein level of Pnc1 increases in the absence of Gas1. Total protein was extracted from WT, *gas1*Δ, *msn2*Δ *msn4*Δ and *gas1*Δ *msn2*Δ *msn4*Δ cells and immunoblotting was performed using a mouse anti-GFP antibody for the detection of GFP-tagged protein. Actin was used as a loading control. The relative ratio of Pnc1 to actin, normalized against that of WT cells, is shown below each lane. Data are representative of at least three independent experiments. (**E**) The loss of Gas1 raises the intracellular NAD^+^ concentration. Values represent the average of three independent experiments, and error bars indicate the standard deviation. Asterisks indicate *P* < 0.05, compared with WT cells (two-tailed Student's *t*-test). (**F**) The association of Sir2 with rDNA is enhanced in the absence of Gas1. The degree of Sir2 binding to four representative regions in the rDNA locus (25S, NTS1, NTS2/18S and 18S regions) was measured using a ChIP assay in the corresponding cells. Values represent the average of three independent experiments, and error bars indicate the standard deviation. Asterisks indicate *P* < 0.05, compared with WT cells (two-tailed Student's *t*-test). (**G**) The loss of Gas1 represses rDNA recombination in an Msn2/4-dependent manner. rDNA recombination is represented by the rate of loss of the *ADE2* marker gene integrated at the rDNA locus in the corresponding cells. Values represent the average of three independent experiments, and error bars indicate the standard deviation. Mean values of the recombination rates for WT, *gas1*Δ, *msn2*Δ *msn4*Δ and *gas1*Δ *msn2*Δ *msn4*Δ cells are 1.94 × 10^−3^, 0.94 × 10^−3^, 1.93 × 10^−3^ and 1.94 × 10^−3^, respectively. Asterisks indicate *P* < 0.05, compared with WT cells (two-tailed Student's *t*-test).

Msn2 and Msn4 are transcription factors that regulate the general stress response of *S. cerevisiae* (33,34). Msn2/4 localize predominantly to the cytoplasm; however, in response to a variety of environmental changes, Msn2/4 localize to the nucleus, where they activate stress-responsive genes (15). A recent study has shown that calorie restriction and TORC1 inhibition stimulate Sir2 activity and rDNA stability by translocalizing Msn2/4 from the cytoplasm to the nucleus, where they enhance the expression of *PNC1*, a gene that encodes a nicotinamidase (12). With this in mind, our observation that the loss of Gas1 enhanced rDNA silencing and reduced rDNA recombination in a Sir2-dependent manner raises the possibility that the translocalization of Msn2/4 and the expression of Pnc1 may be related to Sir2-mediated rDNA stability in *gas1*Δ cells. To investigate this possibility, we performed fluorescence microscopic analysis on *gas1*Δ cells in which the endogenous *MSN2* and *MSN4* genes were modified to produce C-terminal GFP fusion proteins. Remarkably, the loss of Gas1 induced the translocalization of Msn2/4 into the nucleus (Figure 1A). The protein levels of Msn2/4 were not influenced by the loss of Gas1 (data not shown). The *PNC1* promoter contains four stress response elements known as Msn2/Msn4-binding sites (10). To examine whether nuclear-localized Msn2/4 bind to the *PNC1* promoter in *gas1*Δ cells, we performed a ChIP experiment followed by a quantitative real-time PCR assay. The absence of Gas1 increased the binding of Msn2/4 to the *PNC1* promoter (Figure 1B). We next examined the expression level of *PNC1* mRNA in *gas1*Δ cells. As expected, the *PNC1* mRNA level was increased in *gas1*Δ cells (Figure 1C). Consistent with this observation, the protein level of Pnc1 was also increased in *gas1*Δ cells (Figure 1D). Notably, the loss of Gas1 did not increase the Pnc1 level in the absence of Msn2/4 (Figure 1D). These results suggest that the loss of Gas1 induces the expression of Pnc1 in an Msn2/4-dependent manner.

Because Pnc1 converts nicotinamide to nicotinic acid as part of the NAD^+^ salvage pathway (10), it is presumable that an elevated level of Pnc1 may raise the intracellular NAD^+^ concentration in *gas1*Δ cells. To examine this possibility, we measured the intracellular NAD^+^ concentration in *gas1*Δ cells. As shown in Figure 1E, the loss of Gas1 caused a >60% increase in the intracellular NAD^+^ concentration. Previously, it has been shown that an increased Pnc1 level enhances the association of Sir2 with the rDNA region (11). Given the above observation that the loss of Gas1 enhanced the expression of Pnc1 and raised the intracellular NAD^+^ concentration, we next investigated whether increased Pnc1 and NAD^+^ levels lead to enhanced association of Sir2 with the rDNA region in *gas1*Δ cells. We constructed yeast strains in which the endogenous *SIR2* gene was modified to express a C-terminal TAP fusion protein and performed a ChIP assay for four representative regions of the rDNA locus—the 25S, NTS1, NTS2/18S and 18S regions. Consistent with previous observations (6,11), Sir2 bound highly to the NTS1 and NTS2/18S regions (Figure 1F). The association of Sir2 with the rDNA region was further enhanced in *gas1*Δ cells. Interestingly, the loss of Gas1 did not increase the degree of Sir2 binding to rDNA in *msn2*Δ *msn4*Δ cells. This result suggests that the loss of Gas1 enhances the association of Sir2 with rDNA in an Msn2/4-dependent manner. The protein level of Sir2 was not changed in the absence of Gas1 or Msn2/4 (Supplementary Figure S2), indicating that the effect of Gas1 and Msn2/4 on the association of Sir2 with the rDNA region is not due to the altered expression of Sir2. In accordance with the ChIP assay results, the loss of Gas1 did not cause a decrease in the rDNA recombination rate in the absence of Msn2/4 (Figure 1G). Taken together, these results suggest that the loss of Gas1 promotes Sir2-mediated rDNA silencing and rDNA stability by relocalizing Msn2/4 from the cytoplasm to the nucleus, where they stimulate the expression of Pnc1, a protein that degrades nicotinamide, a physiological inhibitor of Sir2. Consequent reduction of nicotinamide and increase of NAD^+^ seem to enhance the recruitment of Sir2 to the rDNA region, thereby promoting rDNA silencing and rDNA stability, as shown previously (11).

### β-1,3-Glucanosyltransferase activity of Gas1 is involved in Sir2-mediated rDNA silencing

**Figure 2.**
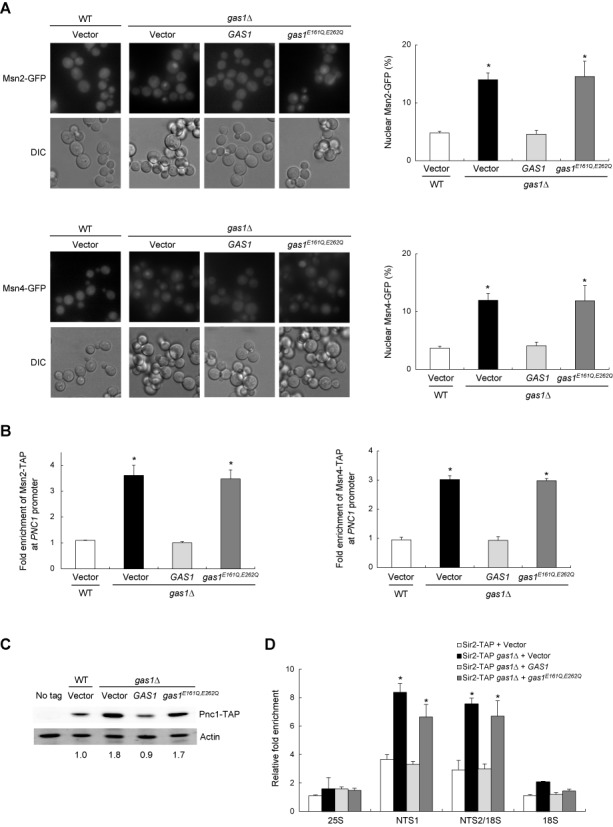
The lack of Gas1 β-1,3-glucanosyltransferase activity promotes nuclear accumulation of Msn2/4 and enhances the association of Msn2/4 with the *PNC1* promoter region. (**A**) The absence of Gas1 β-1,3-glucanosyltransferase activity induces nuclear accumulation of Msn2 (upper panels) and Msn4 (lower panels). Subcellular localization of Msn2- and Msn4-GFP was analyzed by fluorescence microscopy in wild-type (WT) and *gas1*Δ cells containing an empty vector and *gas1*Δ cells expressing WT *GAS1* or *gas1*^*E161Q*, *E262Q*^ on the pRS415 vector (left panels). The percentage of nuclear Msn2- and Msn4-GFP is shown in the right panels. Values represent the average of three independent experiments and at least 100 cells were counted for each determination. Error bars indicate the standard deviation. Asterisks indicate *P* < 0.05, compared with WT cells with an empty vector (two-tailed Student's *t*-test). (**B**) The association of Msn2 (left panel) and Msn4 (right panel) with the *PNC1* promoter region is enhanced in the absence of Gas1 β-1,3-glucanosyltransferase activity. Shown is the degree of association of Msn2 and Msn4 with the *PNC1* promoter region measured using a ChIP assay in the corresponding cells. Values represent the average of three independent experiments and error bars indicate the standard deviation. Asterisks indicate *P* < 0.05, compared with WT cells with an empty vector (two-tailed Student's *t*-test). (**C**) The protein level of Pnc1 increases in the absence of Gas1 β-1,3-glucanosyltransferase activity. Total protein was extracted from the corresponding cells, and immunoblotting was performed using an HRP-conjugated anti-mouse IgG antibody for the detection of TAP-tagged protein. Actin was used as a loading control. The relative ratio of Pnc1 to actin, normalized against that of WT cells, is shown below each lane. Data are representative of at least three independent experiments. (**D**) The association of Sir2 with rDNA is enhanced in the absence of Gas1 β-1,3-glucanosyltransferase activity. The degree of Sir2 binding to four representative regions in the rDNA locus (25S, NTS1, NTS2/18S and 18S regions) was measured using a ChIP assay in the corresponding cells. Values represent the average of three independent experiments, and error bars indicate the standard deviation. Asterisks indicate *P* < 0.05, compared with WT cells with an empty vector (two-tailed Student's *t*-test).

The β-1,3-glucanosyltransferase activity of Gas1 plays a role in the formation of β-1,3-glucanosidic bonds and the crosslinking of cell wall components (20,35). Two amino acid residues, E161 and E262, are critical for the β-1,3-glucanosyltransferase activity of Gas1 (36,37). Koch and Pillus (21) demonstrated that cells carrying the *GAS1* gene with E161Q and E262Q mutations (*gas1^E161Q^*^, *E262Q*^) are defective in telomeric silencing, indicating that the enzymatic activity of Gas1 is necessary for telomeric silencing. However, whether the β-1,3-glucanosyltransferase activity of Gas1 is linked to the Msn2/4–Pnc1–Sir2 pathway of rDNA silencing is unknown. To check this, we analyzed the subcellular localization of Msn2/4 in *gas1^E161Q^*^, *E262Q*^ mutant cells. Like *gas1*Δ cells, *gas1^E161Q^*^, *E262Q*^ cells also exhibited a significant increase in nuclear-localized Msn2/4 (Figure 2A). The protein level of Gas1 was little, if any, changed in cells carrying the *GAS1* gene with E161Q and E262Q mutations (*gas1^E161Q^*^, *E262Q*^) compared with cells carrying wild-type *GAS1* (Supplementary Figure S3). We next examined the degree of Msn2/4 binding to the *PNC1* promoter in *gas1^E161Q^*^, *E262Q*^ cells. As expected, the binding of Msn2/4 to the *PNC1* promoter was significantly increased in *gas1^E161Q^*^, *E262Q*^ cells (Figure 2B). Consistent with this result, cells with Gas1^E161Q, E262Q^ exhibited increased expression of Pnc1 compared with cells with wild-type Gas1 (Figure 2C). Moreover, a ChIP assay to measure the association of Sir2 with the rDNA region revealed that cells with Gas1^E161Q, E262Q^ exhibited significantly enhanced association of Sir2 with the rDNA region compared with cells with wild-type Gas1 (Figure 2D). These results suggest that the *gas1^E161Q, E262Q^* mutant defective in β-1,3-glucanosyltransferase activity and the *gas1*Δ knockout mutant exhibit similar effects on the nuclear localization of Msn2/4, the binding of Msn2/4 to the *PNC1* promoter, the expression of Pnc1, and the association of Sir2 with rDNA.

**Figure 3.**
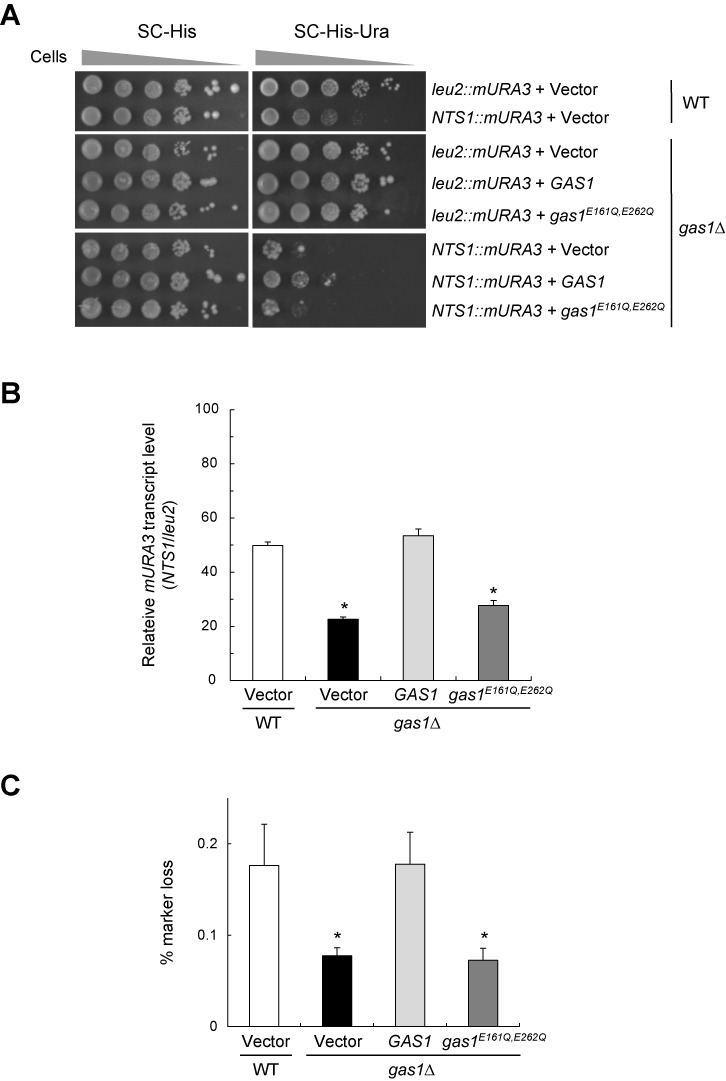
The lack of Gas1 β-1,3-glucanosyltransferase activity promotes rDNA silencing and rDNA stability. (**A**) The lack of Gas1 β-1,3-glucanosyltransferase activity increases silencing of the *mURA3* reporter gene at the rDNA region. Silencing within the rDNA region was assessed by monitoring the growth of cells (10-fold serial dilutions) plated on SC medium without histidine and uracil. SC without histidine medium was used as a plating control. The spot assay was performed with WT and *gas1*Δ cells containing an empty vector and *gas1*Δ cells expressing WT *GAS1* and *gas1*^*E161Q*, *E262Q*^ on the pRS413 vector. (**B**) The absence of Gas1 β-1,3-glucanosyltransferase activity induces transcriptional silencing of the *mURA3* reporter gene at the rDNA locus. Total RNA was extracted from the corresponding cells and quantitative real-time reverse transcription-PCR analysis was performed as in Supplementary Figure S1B. The values were the mean of three independent experiments and error bar indicates the standard deviation. Asterisks indicate *P* < 0.05, compared with WT cells with an empty vector (two-tailed Student's *t*-test). (**C**) The lack of Gas1 β-1,3-glucanosyltransferase activity represses rDNA recombination. rDNA recombination is represented by the rate of loss of the *ADE2* marker gene integrated at the rDNA locus in the corresponding cells. Values represent the average of three independent experiments, and error bars indicate the standard deviation. Mean values of the recombination rates for WT + vector, *gas1*Δ + vector, *gas1*Δ + *GAS1* and *gas1*Δ + *gas1^E161Q, E262Q^* cells are 1.76 × 10^−3^, 0.76 × 10^−3^, 1.78 × 10^−3^ and 0.73 × 10^−3^, respectively. Asterisks indicate *P* < 0.05, compared with WT cells with an empty vector (two-tailed Student's *t*-test).

Given the above observations, we next examined whether the loss of Gas1 enzymatic activity affects transcriptional silencing at the rDNA region. We performed an rDNA silencing assay using the *mURA3* reporter gene integrated at the rDNA locus. In wild-type cells carrying an empty vector, the reporter gene was efficiently silenced at the rDNA region, as indicated by poor growth on medium lacking uracil (Figure 3A). As expected, silencing of *mURA3* at the rDNA region was further enhanced in *gas1*Δ cells with an empty vector. Notably, *gas1*Δ cells complemented with *gas1^E161Q^*^, *E262Q*^ also exhibited enhanced silencing of *mURA3* at the rDNA region, while *gas1*Δ cells complemented with wild-type *GAS1* did not. Consistent with these results, a real-time reverse transcription-PCR analysis revealed that the transcript level of the Pol II-transcribed *mURA3* gene integrated inside the rDNA array was significantly lower in *gas1*Δ cells complemented with *gas1^E161Q^*^, *E262Q*^ than in those complemented with wild-type *GAS1* (Figure 3B). In addition, cells devoid of Gas1 β-1,3-glucanosyltransferase activity exhibited a >50% decrease in the rate of rDNA recombination (Figure 3C). Collectively, these observations suggest that the β-1,3-glucanosyltransferase activity of Gas1 is involved in Sir2-mediated transcriptional silencing at the rDNA region and that its loss contributes to increased rDNA stability through the reduction of rDNA recombination.

### Congo red treatment promotes rDNA silencing and rDNA stability in a Sir2-dependent manner

The above result that a defect in the β-1,3-glucanosyltransferase activity of Gas1 enhances Sir2-mediated rDNA silencing raises the question of whether the effect of Gas1 on rDNA silencing is related to its role in cell wall integrity. In *S. cerevisiae*, cell wall damage induces up-regulation of a variety of genes, and several transcription factors, including Msn2/4, are involved in this process (38,39). In particular, Garcia *et al.* (39) noticed that the transcriptomic profile of the *gas1*Δ mutant is similar to that identified in response to Congo red, a β-1,3-glucan-binding dye that causes cell wall damage. Given these observations, it seems likely that Congo red treatment and the *gas1*Δ mutation may have similar effects on Sir2-mediated rDNA stability. To investigate this possible effect, we first examined the association of Msn2/4 with the *PNC1* promoter region in the presence of Congo red. Msn2/4 exhibited a significantly enhanced association with the *PNC1* promoter region in cells treated with Congo red (Supplementary Figure S4A). Next, we examined the expression of Pnc1 and the association of Sir2 with the rDNA region in the presence of Congo red. As expected, like the *gas1*Δ mutation, Congo red treatment induced the expression of Pnc1 (Supplementary Figure S4B) and increased the association of Sir2 with the rDNA region (Supplementary Figure S4C). Consistent with these observations, cells treated with Congo red exhibited enhanced silencing of *mURA3* at the rDNA region compared with control cells (Supplementary Figure S4D). However, the loss of Sir2 abolished silencing of *mURA3* at the rDNA region in Congo red-treated cells, indicating that the effect of Congo red on transcriptional silencing at the rDNA region is dependent on the presence of Sir2. A real-time reverse transcription-PCR analysis to measure the transcript levels of the Pol II-transcribed *mURA3* gene integrated inside or outside the rDNA array confirmed the above spot assay result (Supplementary Figure S4E). In addition, wild-type cells showed a significant decrease in the rDNA recombination rate under Congo red treatment, whereas *sir2*Δ cells did not (Supplementary Figure S4F). Taken together, these results demonstrate that, like the *gas1*Δ or *gas1^E161Q^*^, *E262Q*^ mutation, Congo red treatment enhances transcriptional silencing at the rDNA region and promotes rDNA stability in a Sir2-dependent manner, suggesting that the enhancement of Sir2-mediated rDNA silencing as a result of Gas1 dysfunction is related to the role of Gas1 in cell wall integrity.

We next examined whether other agents that cause cell wall stress affect transcriptional silencing at the rDNA region. Calcofluor white, a chitin antagonist that interferes with cell wall biogenesis, did not promote rDNA silencing (Supplementary Figure S5A and S5B) and did not significantly reduce the rDNA recombination rate (Supplementary Figure S5C). In addition, SDS, vanadate and caffeine, which indirectly weaken the cell wall, had little, if any, effect on rDNA silencing (Supplementary Figure S5D). These results suggest that only direct interruption of the formation and maintenance of β-1,3-glucan affects rDNA silencing and rDNA stability.

### Gas1 paralogs are not involved in rDNA silencing

In addition to Gas1, many enzymes function in the formation and maintenance of the cell wall. The *GAS* multigene family of *S. cerevisiae* is composed of five paralogs (*GAS1* to *GAS5*). *GAS1* encodes a cell wall-bound β-1,3-glucanosyltransferase functioning as a β-1,3-glucan elongase and is required for proper cell wall assembly and maintenance during vegetative growth (19). *GAS5* is also expressed during vegetative growth. *GAS2* and *GAS4* are expressed exclusively during sporulation and are required for spore wall assembly (40). *GAS3* is the lowest expressed paralog of the *GAS* family during vegetative growth, and its mRNA increases during sporulation (41). *BGL2* encodes an endo-β-1,3-glucanase involved in cell wall construction and maintenance (42). To determine whether these proteins that contribute enzymatically to the cell wall have a functional relationship with rDNA silencing, as was seen for Gas1, deletions of the corresponding genes were constructed with the *mURA3* silencing reporter gene and the *ADE2* recombination marker gene integrated at the rDNA locus. Intriguingly, unlike *gas1*Δ cells, these mutants did not show enhanced silencing of *mURA3* at the rDNA region (Supplementary Figure S6A) nor did they exhibit a significant reduction in the rDNA recombination rate compared with wild-type cells (Supplementary Figure S6B). These observations suggest that a functional relationship with rDNA silencing is not a general characteristic of proteins involved in the formation and maintenance of the cell wall but rather is specific to Gas1.

### Gas1 controls PKA activity in a cell wall integrity MAP kinase Slt2-dependent manner

**Figure 4.**
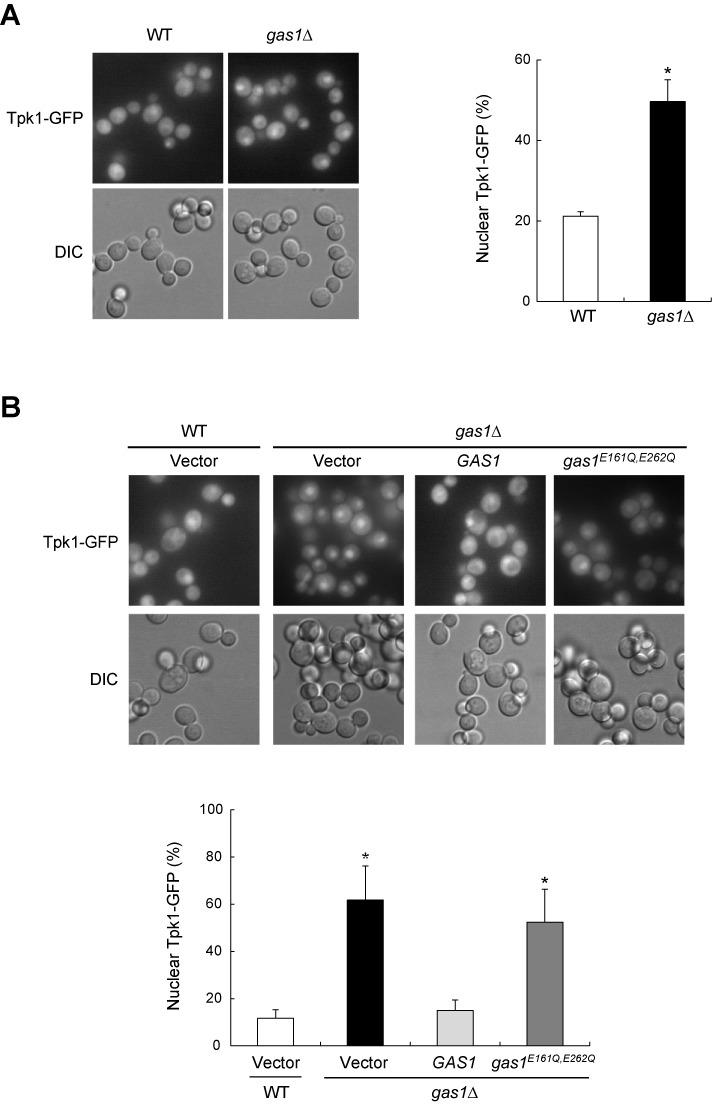
The loss of Gas1 induces nuclear accumulation of the PKA catalytic subunit Tpk1. (**A**) The absence of Gas1 promotes nuclear accumulation of Tpk1. Subcellular localization of Tpk1-GFP was analyzed by fluorescence microscopy in wild-type (WT) and *gas1*Δ cells (left panel). The percentage of nuclear Tpk1-GFP is shown in the right panel. Values represent the average of three independent experiments and at least 100 cells were counted for each determination. Error bars indicate the standard deviation. Asterisks indicate *P* < 0.05, compared with WT cells (two-tailed Student's *t*-test). (**B**) The absence of Gas1 β-1,3-glucanosyltransferase activity induces nuclear accumulation of Tpk1. Subcellular localization of Tpk1-GFP was analyzed by fluorescence microscopy in WT and *gas1*Δ cells containing an empty vector and *gas1*Δ cells expressing WT *GAS1* or *gas1*^*E161Q*, *E262Q*^ on the pRS415 vector (upper panel). The percentage of nuclear Tpk1-GFP is shown in the lower panel. Values represent the average of three independent experiments and at least 100 cells were counted for each determination. Error bars indicate the standard deviation. Asterisks indicate *P* < 0.05, compared with WT cells with an empty vector (two-tailed Student's *t*-test).

Previous studies have shown that nuclear Msn2/4 are activated by dephosphorylation and are inhibited by phosphorylation (15,43). PKA activity influences Msn2 phosphorylation/dephosphorylation and regulates the intracellular distribution and intracellular function of Msn2 (13,15,44). High PKA activity promotes the PKA phosphorylation cascade, including Msn2 phosphorylation, and inhibits the import of Msn2 into the nucleus, while low PKA activity stimulates Msn2 dephosphorylation and allows entry of Msn2 into the nucleus (13,16,45). Inactivation of PKA results in a nuclear accumulation of the PKA catalytic subunit, Tpk1, indicating that catalytically inactive PKA resides in the nucleus (46–48). Above, we showed that the loss of Gas1 induces the translocalization of Msn2/4 into the nucleus (Figure 1A). To uncover how Gas1 affects the subcellular localization of Msn2/4, we examined the Tpk1 localization in *gas1*Δ cells. In wild-type cells, Tpk1 was generally evenly distributed between the nucleus and the cytoplasm, while the loss of Gas1 induced a nuclear accumulation of Tpk1 (Figure 4A). The protein level of Tpk1 was not affected by the loss of Gas1 (data not shown). *gas1^E161Q, E262Q^* cells devoid of Gas1 β-1,3-glucanosyltransferase activity also exhibited increased nuclear accumulation of Tpk1 compared with control cells (Figure 4B). These results suggest that Gas1 contributes to the maintenance of PKA activity and that the translocalization of Msn2/4 into the nucleus observed in *gas1*Δ or *gas1^E161Q, E262Q^* cells is due to lowered PKA activity.

**Figure 5.**
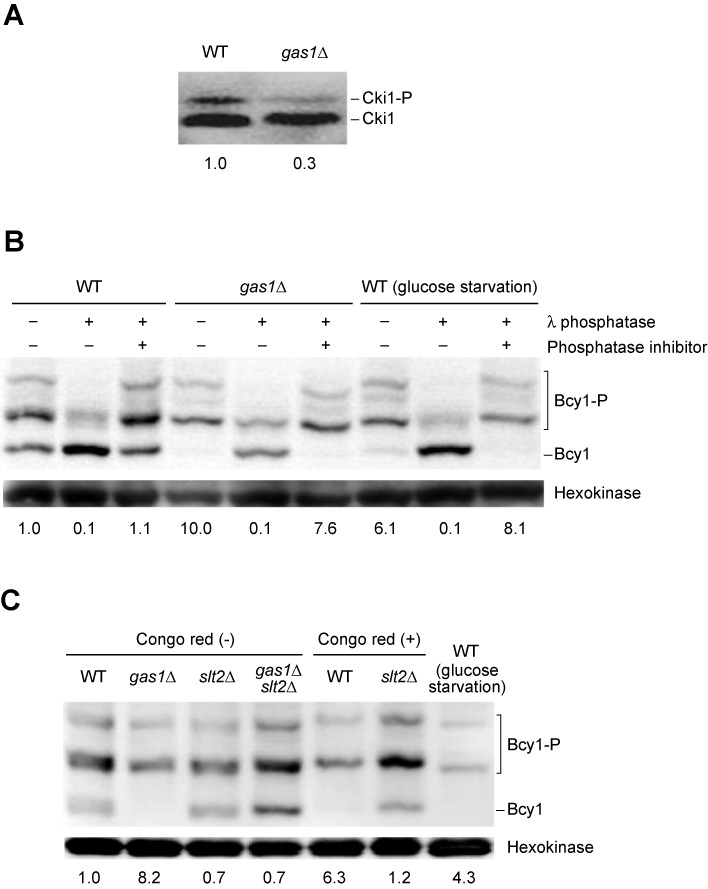
Gas1 controls PKA activity in an Slt2-dependent manner. (**A**) The absence of Gas1 decreases the *in vivo* activity of PKA. Total protein was extracted from wild-type (WT) and *gas1*Δ cells harboring pRS423-pr*^CUP^-6×MYC-cki1^2^*^−*200(S125*^*^/130A)^*, and immunoblotting was performed using a mouse anti-Myc antibody. The relative ratio of phosphorylated (Cki1-P) to unphosphorylated (Cki1) forms of Cki1, normalized against that of WT cells, is shown below each lane. Data are representative of at least three independent experiments. (**B**) The loss of Gas1 increases phosphorylation of Bcy1. Total protein was extracted from WT and *gas1*Δ cells with N-terminally HA-tagged Bcy1 and run on an SDS-polyacrylamide gel containing 20 μM Phos-tag. Immunoblotting was performed using an HRP-conjugated anti-HA antibody for the detection of HA-Bcy1. Hexokinase was used as a loading control. The relative ratio of phosphorylated (Bcy1-P) to unphosphorylated (Bcy1) forms of Bcy1, normalized against that of untreated WT cells, is shown below each lane. Data are representative of at least three independent experiments. (**C**) The loss of Gas1 and Congo red treatment increases phosphorylation of Bcy1 in an Slt2-dependent manner. Total protein was extracted from WT, *gas1*Δ, *slt2*Δ and *gas1*Δ *slt2*Δ cells with N-terminally HA-tagged Bcy1 and run on an SDS-polyacrylamide gel containing 20 μM Phos-tag (lanes 1–4). Protein extracts from WT and *slt2*Δ cells treated with 100 μg/ml Congo red for 1 h were also run on the same gel (lanes 5 and 6). Immunoblotting was performed using an HRP-conjugated anti-HA antibody for the detection of HA-Bcy1. Hexokinase was used as a loading control. The relative ratio of phosphorylated (Bcy1-P) to unphosphorylated (Bcy1) forms of Bcy1, normalized against that of untreated WT cells, is shown below each lane. Data are representative of at least three independent experiments.

To confirm that Gas1 is required for the maintenance of PKA activity, we measured the *in vivo* activity of PKA in *gas1*Δ cells using a PKA substrate reporter, which was created from a native substrate protein, Cki1 (23). This PKA substrate reporter contains the first 200 amino acids of Cki1 and possesses mutations at two known protein kinase C sites (S125A and S130A) making its phosphorylation exclusively dependent on PKA. We detected PKA-dependent phosphorylation of the Cki1 reporter by a mobility shift in SDS-polyacrylamide gel electrophoresis analysis and quantified the ratio of phosphorylated and unphosphorylated forms as a measure for the *in vivo* activity of PKA in cells as previously described (24). As expected, we observed a considerable decrease in the phosphorylated Cki1-P form in *gas1*Δ cells compared with wild-type cells (Figure 5A). Consistent with this result, cells with Gas1^E161Q, E262Q^ exhibited a considerable decrease in the phosphorylated Cki1-P form compared with cells with wild-type Gas1 (Supplementary Figure S7A). Moreover, Congo red treatment also led to a decrease in the phosphorylated Cki1-P form (Supplementary Figure S7B). These observations demonstrate that the *in vivo* activity of PKA is lowered not only in *gas1*Δ cells but also in *gas1^E161Q, E262Q^* cells and in cells treated with Congo red. Collectively, these results suggest that Gas1 acts as a positive regulator of PKA activity.

The PKA regulatory subunit Bcy1 is highly phosphorylated upon glucose starvation or TORC1 inactivation, and this phosphorylation appears to be important for nuclear localization of PKA, suggesting that Bcy1 phosphorylation leads to Bcy1 activation and PKA inactivation (49–51). To further investigate whether Gas1 affects PKA activity via Bcy1 phosphorylation, we measured the electrophoretic mobility of Bcy1 in *gas1*Δ cells using a phosphate-binding tag (Phos-tag)-based analysis for better separation of phosphorylated Bcy1. We observed that, as in the case of glucose starvation, the loss of Gas1 resulted in an increase in Bcy1 phosphorylation (Figure 5B), indicating that Bcy1 is highly phosphorylated in the absence of Gas1. This result supports the above notion that the loss of Gas1 decreases PKA activity and thereby leads to the translocalization of Msn2/4 into the nucleus.

Our observations that the loss of Gas1 reduces PKA activity and leads to the translocalization of Msn2/4 into the nucleus raise the possibility that PKA-dependent phosphorylation status of Msn2/4 may be related to rDNA silencing in *gas1*Δ cells. Previous studies have shown that four amino acid residues (S582, S620, S625 and S633) are PKA-dependent phosphorylation sites of Msn2 (15,43). In an rDNA silencing assay using the *mURA3* reporter gene integrated inside or outside the rDNA locus, *gas1*Δ *msn2*Δ cells carrying an empty vector exhibited silencing of the *mURA3* reporter gene at the rDNA region, as indicated by poor growth on medium lacking uracil (Supplementary Figure S8). As expected, silencing of *mURA3* at the rDNA region was further enhanced in *gas1*Δ *msn2*Δ cells complemented with wild-type *MSN2*. Notably, silencing of *mURA3* at the rDNA region was significantly lower in *gas1*Δ *msn2*Δ cells complemented with a phospho-mimic mutant of *MSN2* (*msn2^S582D, S620D, S625D, S633D^*) than in those complemented with wild-type *MSN2.* This result suggests that PKA-dependent phosphorylation/dephosphorylation of Msn2 contributes to the maintenance of rDNA silencing.

The cell wall integrity MAP kinase cascade, which consists of Bck1, Mkk1/2 and Slt2, is activated by Pkc1 (52,53). Several reports have linked the cell wall integrity pathway to Slt2 phosphorylation; stimulation of the cell wall integrity pathway (e.g. the loss of Gas1) results in phosphorylation and activation of the MAP kinase Slt2 (38,54,55). Recently, it has been shown that the activated form of Slt2, which is formed by perturbation of the cell wall, directly phosphorylates Bcy1 (51). These observations raise the possibility that the loss of Gas1 may induce phosphorylation of Bcy1 in an Slt2-dependent manner. To investigate this possibility, we examined whether the absence of Slt2 affects the electrophoretic mobility of Bcy1 in *gas1*Δ cells. Remarkably, the deletion of *SLT2* abolished the increase in Bcy1 phosphorylation induced by the *gas1*Δ mutation (Figure 5C), suggesting that Slt2 phosphorylates Bcy1 in *gas1*Δ cells. We also observed that Congo red treatment led to an increase in Bcy1 phosphorylation and that the loss of Slt2 abolished the increase in Bcy1 phosphorylation induced by Congo red treatment. This result is consistent with our above finding that Congo red treatment and the *gas1*Δ mutation have similar effects on Sir2-mediated rDNA stability. Taken together, all our results suggest that the loss of Gas1 lowers PKA activity via Bcy1 phosphorylation in a cell wall integrity MAP kinase Slt2-dependent manner and leads to the translocalization of Msn2/4 into the nucleus. It seems that nuclear-localized Msn2/4 stimulate the expression of Pnc1 and consequent reduction of nicotinamide and increase of NAD^+^ enhance the association of Sir2 with the rDNA region, thereby promoting rDNA silencing and rDNA stability.

## DISCUSSION

The β-1,3-glucanosyltransferase Gas1 plays a role in the formation and maintenance of β-1,3-glucan, which is the major polysaccharide of the cell wall (18). Gas1 provides the activity required to elongate and rearrange β-1,3-glucan side chains after β-1,3-glucan is synthesized. In this study, we demonstrate that Gas1 participates in rDNA silencing and rDNA stability in *S. cerevisiae*. The loss of Gas1 induces Sir2-mediated rDNA stability by the translocalization of Msn2/4 from the cytoplasm to the nucleus. Once in the nucleus, Msn2/4 stimulate the expression of *PNC1*, which encodes a nicotinamidase that is a Sir2 activator. The roles of Gas1 in rDNA silencing and rDNA stability seem to be mediated by its β-1,3-glucanosyltransferase activity, given that cells with the *gas1^E161Q, E262Q^* mutation, which are defective in β-1,3-glucanosyltransferase activity, and the *gas1*Δ knockout cells exhibit similar effects on rDNA silencing and rDNA stability. In addition, we observed that a cell wall-damaging agent, Congo red, plays a positive role in rDNA stability in a Sir2-dependent manner. Our data suggest that the enhancement of Sir2-mediated rDNA silencing as a result of Gas1 dysfunction is related to the role of Gas1 in cell wall integrity. This finding is somewhat different from that of Koch and Pillus (21), who proposed that Gas1 may add a carbohydrate moiety to Sir2 or other factors that interact with Sir2, thereby altering the enzymatic activity of Sir2 and distinctly affecting the transcriptional silencing of different chromatin loci. Further studies are needed to establish whether the effect of Gas1 on rDNA silencing is mainly due to its role in cell wall integrity or to its role in carbohydrate modification of Sir2 or Sir2-interacting proteins.

Intriguingly, among the *GAS* multigene family of *S. cerevisiae* (*GAS1* to *GAS5*), only *GAS1* appears to have a functional relationship with rDNA silencing. Consistent with this observation, the telomeric silencing function of *GAS1* is not shared with other *GAS* family genes (21). It is plausible that *GAS2* and *GAS4* do not affect rDNA silencing because they are expressed exclusively during sporulation and are required for spore wall assembly (40). Among the *GAS* family members that are expressed during vegetative growth (*GAS1*, *GAS3* and *GAS5*), *GAS1* is the most highly expressed paralog; the transcripts of *GAS1* and *GAS5* are 34 and 8 times more abundant, respectively, than the transcript of *GAS3* (41). Thus, it is likely that Gas1 provides the main β-1,3-glucanosyltransferase activity of *S. cerevisiae* during vegetative growth. This notion may explain why *GAS1* is the only *GAS* multigene family member involved in rDNA silencing.

Transcriptional silencing phenotypes of *gas1*Δ cells are locus-specific; the loss of Gas1 causes reduced silencing at telomeres but enhanced silencing at rDNA (21). The locus-specific silencing phenotypes have also been observed in several mutants of the Slt2 MAP kinase pathway (27). In yeast, it is believed that silencing is mediated by four Sir proteins (Sir1, Sir2, Sir3 and Sir4) and that the size of the Sir protein pool determines the extent of silencing. It has been reported that rDNA silencing is limited by the availability of Sir2 (56). Given that the association of Sir2 with the rDNA region is enhanced in *gas1*Δ cells (Figure 1F) while the protein level of Sir2 is not changed (Supplementary Figure S2), it is likely that the loss of Gas1 leads to the redistribution of Sir2 from telomeres to the rDNA region. Telomeres and rDNA are thought to be reservoirs for Sir2 that can be redistributed under specific conditions. The molecular mechanisms underlying the redistribution of Sir2 in *gas1*Δ cells remain to be elucidated.

We observed that transcriptional silencing at the rDNA region is affected by β-1,3-glucan-binding dye Congo red but not by other cell wall-perturbing agents such as Calcofluor white, SDS, vanadate and caffeine (Supplementary Figure S5). Given that Calcofluor white, SDS, vanadate and caffeine also induce phosphorylation and activation of Slt2 (54,57,58), it is intriguing that they do not affect rDNA silencing. Calcofluor white is a chitin antagonist and has a much weaker binding affinity toward β-1,3-glucan than Congo red (59). SDS, vanadate and caffeine indirectly weaken the cell wall. Presumably, among several cell wall-perturbing agents, those that directly interrupt the formation and maintenance of β-1,3-glucan (e.g. Congo red) can induce Slt2 activation strong enough to be effective in rDNA silencing.

PKA is a part of the RAS-cAMP signaling cascade that controls various growth processes, including translation, ribosome biogenesis, autophagy, stress responses, glucose metabolism and lifespan (60). Slt2 is a MAP kinase in the cell wall integrity pathway that is activated in response to several stresses (52). Our observation that Slt2 phosphorylates and activates the PKA regulatory subunit Bcy1 in *gas1*Δ cells corroborates the relationship between the cell wall integrity pathway and the PKA pathway. Consistent with this finding, previous studies have shown that the components of the cell wall integrity pathway genetically interact with those of the PKA pathway (55,61). Considering these previous studies and our findings that Gas1 controls PKA through Slt2 and that PKA eventually regulates Sir2-mediated rDNA silencing, we propose a role of the cell wall integrity pathway in the regulation of PKA activity and transcriptional silencing.

Given that Sir2-mediated rDNA silencing inhibits the formation of extrachromosomal rDNA circles, which are toxic to cells and induce replicative aging in *S. cerevisiae* (2), it is likely that enhanced rDNA silencing as a result of Gas1 dysfunction may lead to an increase in replicative lifespan. To examine this possibility, we tried to measure the replicative lifespan of *gas1*Δ cells. However, we could not obtain evidence for an increase in the replicative lifespan of the *gas1*Δ cells because they exhibited severe aggregation and were not amenable to conventional micromanipulation (data not shown). It will be interesting to further investigate the role of Gas1 in the replicative lifespan of yeast; such research may provide new insights into the regulatory mechanisms of cell aging in yeast.

## SUPPLEMENTARY DATA

Supplementary Data are available at NAR Online.

SUPPLEMENTARY DATA
