# Supplementary material for: The β-1,3-glucanosyltransferase Gas1 regulates Sir2-mediated rDNA stability in Saccharomyces cerevisiae
Source: Nucleic Acids Res. 2014 Jun 30;42(13):8486–99. doi: 10.1093/nar/gku570 (PMC4117787; doi:10.1093/nar/gku570)
Supplement: SUPPLEMENTARY DATA [file supp_42_13_8486__index.html]

The β-1,3-glucanosyltransferase Gas1 regulates Sir2-mediated rDNA stability in Saccharomyces cerevisiae — The β-1,3-glucanosyltransferase Gas1 regulates Sir2-mediated rDNA stability in Saccharomyces cerevisiae — SUPPLEMENTARY DATA 

# The β-1,3-glucanosyltransferase Gas1 regulates Sir2-mediated rDNA stability in *Saccharomyces cerevisiae*

## SUPPLEMENTARY DATA

**Files in this Data Supplement:**

- SUPPLEMENTARY DATA
