# Supplementary material for: The β-1,3-glucanosyltransferase Gas1 regulates Sir2-mediated rDNA stability in Saccharomyces cerevisiae
Source: Nucleic Acids Res. 2014 Jun 30;42(13):8486–99. doi: 10.1093/nar/gku570 (PMC4117787; doi:10.1093/nar/gku570)

Supplementary Figure Legends

Supplementary Figure 1. The loss of Gas1 enhances rDNA silencing and rDNA stability in the presence of Sir2. (A) The absence of Gas1 increases rDNA silencing. Silencing within rDNA was assessed by monitoring the growth of cells (10-fold serial dilutions) plated on SC medium without uracil. SC medium was used as a plating control. (B) The loss of Gas1 promotes transcriptional silencing of the *mURA3* reporter gene at the rDNA locus in a Sir2-dependent manner. Total RNA was extracted from wild-type (WT), *gas1*Δ, *sir2*Δ, and *gas1*Δ *sir2*Δ cells. Quantitative real-time reverse transcription-PCR analysis was performed to measure the transcript levels of the *mURA3* reporter gene inserted inside (*RDN1-NTS1*::*mURA3*) or outside the rDNA array (*leu2*::*mURA3*). Amplification efficiencies were validated and normalized against *ACT1*. Relative *mURA3* transcript levels were calculated as the ratio of the normalized transcript level of the *mURA3* reporter gene inside the NTS1 region to that outside the rDNA array. The values were the mean of three independent experiments and error bar indicates standard deviations. Asterisks indicate $P<0.05$, compared with WT cells (two-tailed Student's *t*-test). (C) The loss of Gas1 represses rDNA recombination in a Sir2-dependent manner. rDNA recombination is represented by the rate of loss of the *ADE2* marker gene integrated at the rDNA locus in WT, *gas1*Δ, *sir2*Δ, and *gas1*Δ *sir2*Δ cells. Values represent the average of three independent experiments, and error bars indicate the standard deviation. Mean values of the recombination rates for WT, *gas1*Δ, *sir2*Δ, and *gas1*Δ *sir2*Δ cells are 1.53×10^{-3}, 0.57×10^{-3}, 4.68×10^{-3}, and 4.84×10^{-3}, respectively. Asterisks indicate $P<0.05$, compared with WT cells (two-tailed Student's *t*-test).

Supplementary Figure S2. The protein level of Sir2 is not changed in the absence of Gas1 or Msn2/4. Total protein was extracted from wild-type (WT), *gas1*Δ, *msn2*Δ *msn4*Δ, and *gas1*Δ *msn2*Δ *msn4*Δ cells, and immunoblotting was performed using an HRP-conjugated anti-mouse IgG antibody for the detection of TAP-tagged protein. Actin was used as a loading control. The relative ratio of Sir2 to actin, normalized against that of WT cells, is shown below each lane. Data are representative of at least three independent experiments.

Supplementary Figure S3. The protein level of Gas1 is not changed in the lack of Gas1 β-1,3-glucanosyltransferase activity. Total protein was extracted from WT and *gas1*Δ cells containing an empty vector and *gas1*Δ cells expressing WT *GAS1* and *gas1* $^{E161Q,\ E262Q}$ on the

pRS413 vector, and immunoblotting was performed using a mouse anti-GFP antibody for the detection of GFP-tagged protein. Hexokinase was used as a loading control. The relative ratio of Gas1 to hexokinase, normalized against that of WT cells, is shown below each lane. Data are representative of at least three independent experiments.

Supplementary Figure S4. Congo red treatment promotes Sir2-mediated rDNA silencing. (A) The association of Msn2/4 with the *PNC1* promoter region is enhanced under Congo red treatment. The degree of association of Msn2-TAP (left panel) and Msn4-TAP (right panel) with the *PNC1* promoter region was measured using a ChIP assay with or without treatment with 100 μg/ml Congo red for 1 h. Cells without Congo red treatment were used as a control. Values represent the average of three independent experiments, and error bars indicate the standard deviation. Asterisks indicate $P<0.05$, compared with untreated control cells (two-tailed Student's *t*-test). (B) The protein level of Pnc1 increases under Congo red treatment. Total protein was extracted from cells with or without treatment with 100 μg/ml Congo red for 1 h, and immunoblotting was performed using an HRP-conjugated anti-mouse IgG antibody for the detection of TAP-tagged protein. Actin was used as a loading control. The relative ratio of Pnc1 to actin, normalized against that of untreated control cells, is shown below each lane. Data are representative of at least three independent experiments. (C) The association of Sir2 with rDNA is enhanced under Congo red treatment. The degree of Sir2 binding to four representative regions in the rDNA locus (25S, NTS1, NTS2/18S, and 18S regions) was measured using a ChIP assay with or without treatment with 100 μg/ml Congo red for 1 h. Values represent the average of three independent experiments, and error bars indicate the standard deviation. Asterisks indicate $P<0.05$, compared with untreated control cells (two-tailed Student's *t*-test). (D) Congo red increases rDNA silencing in the presence of Sir2. Silencing within the rDNA region was assessed by monitoring the growth of cells (10-fold serial dilutions) plated on SC medium without uracil in the presence or absence of 100 μg/ml Congo red. SC medium was used as a plating control. (E) Congo red promotes transcriptional silencing of the *mURA3* reporter gene at the rDNA locus in a Sir2-dependent manner. Total RNA was extracted from WT and *sir2*Δ cells with or without treatment with 100 μg/ml Congo red for 1 h. Quantitative real-time reverse transcription-PCR analysis was performed as in Supplementary Figure 1B. The values were the mean of three independent experiments and error bar indicates standard deviations. Asterisks indicate $P<0.05$, compared with untreated WT cells (two-tailed Student's *t*-test). (F) Congo red suppresses rDNA

recombination in a Sir2-dependent manner. rDNA recombination is represented by the rate of loss of the *ADE2* marker gene integrated at the rDNA locus in WT and *sir2*Δ cells plated on SC medium with or without 100 μg/ml Congo red. Values represent the average of three independent experiments, and error bars indicate the standard deviation. Mean values of the recombination rates for WT (control), WT (Congo red), *sir2*Δ (control), and *sir2*Δ (Congo red) cells are 1.45×10^{-3}, 0.64×10^{-3}, 4.68×10^{-3}, and 4.21×10^{-3}, respectively. Asterisks indicate $P<0.05$, compared with untreated WT cells (two-tailed Student's *t*-test).

Supplementary Figure S5. Cell wall stress agents, such as calcofluor white, SDS, vanadate, and caffeine, do not affect rDNA silencing. (A) Calcofluor white (CFW) does not promote rDNA silencing. Silencing within the rDNA region was assessed by monitoring the growth of cells (10-fold serial dilutions) plated on SC medium without uracil in the presence or absence of 50 μg/ml CFW. SC medium was used as a plating control. (B) CFW does not promote transcriptional silencing of the *mURA3* reporter gene at the rDNA locus. Total RNA was extracted from wild-type and *sir2*Δ cells with or without treatment with 50 μg/ml CFW for 1 h. Quantitative real-time reverse transcription-PCR analysis was performed to measure the transcript levels of the *mURA3* reporter gene inserted inside (*RDN1-NTS1*::*mURA3*) or outside the rDNA array (*leu2*::*mURA3*). Values represent the average of three independent experiments, and error bars indicate the standard deviation. (C) CFW does not promote rDNA stability. rDNA recombination is represented by the rate of loss of the *ADE2* marker gene integrated at the rDNA locus in wild-type and *sir2*Δ cells plated on SC medium with or without 50 μg/ml CFW. Values represent the average of three independent experiments, and error bars indicate the standard deviation. Mean values of the recombination rates for WT (control), WT (CFW), *sir2*Δ (control), and *sir2*Δ (CFW) cells are 1.53×10^{-3}, 1.39×10^{-3}, 4.68×10^{-3}, and 4.62×10^{-3}, respectively. (D) SDS, vanadate, and caffeine do not promote transcriptional silencing of the *mURA3* reporter gene at the rDNA locus. Total RNA was extracted from cells with or without treatment with 0.01% SDS, 5 mM vanadate, or 12 mM caffeine for 1 h. Quantitative real-time reverse transcription-PCR analysis was performed as described above. Values represent the average of three independent experiments, and error bars indicate the standard deviation.

Supplementary Figure S6. Gas1 paralogs are not involved in rDNA silencing. (A) The absence of Gas1 paralogs does not increase rDNA silencing. The spot assay was performed in

wild-type (WT), *gas1*Δ, *gas2*Δ, *gas3*Δ, *gas4*Δ, *gas5*Δ, and *bgl2*Δ cells. Silencing within rDNA was assessed by monitoring the growth of cells (10-fold serial dilutions) plated on SC medium without uracil. SC medium was used as a plating control. (B) The absence of Gas1 paralogs does not significantly contribute to rDNA stability. rDNA recombination is represented by the rate of loss of the *ADE2* marker gene integrated at the rDNA locus in WT, *gas1*Δ, *gas2*Δ, *gas3*Δ, *gas4*Δ, *gas5*Δ, and *bgl2*Δ cells. Values represent the average of three independent experiments, and error bars indicate the standard deviation. Mean values of the recombination rates for WT, *gas1*Δ, *gas2*Δ, *gas3*Δ, *gas4*Δ, *gas5*Δ, and *bgl2*Δ cells are 1.48×10^{-3}, 0.40×10^{-3}, 2.07×10^{-3}, 1.28×10^{-3}, 1.01×10^{-3}, 1.05×10^{-3}, and 1.35×10^{-3}, respectively. Asterisks indicate $P<0.05$, compared with WT cells (two-tailed Student's *t*-test).

Supplementary Figure S7. The lack of Gas1 β-1,3-glucanosyltransferase activity and the treatment of Congo red decrease the *in vivo* activity of PKA. (A) The absence of Gas1 β-1,3-glucanosyltransferase activity decreases the *in vivo* activity of PKA. Total protein was extracted from WT and *gas1*Δ cells harboring pRS423-pr^{CUP}-*6×MYC-cki1*$^{2-200(S125/130A)}$ and containing an empty, WT *GAS1* and *gas1*$^{E161Q, E262Q}$ on the pRS415 vector. Immunoblotting was performed using a mouse anti-Myc antibody. The relative ratio of phosphorylated (Cki1-P) to non-phosphorylated (Cki1) forms of Cki1, normalized against that of WT cells, is shown below each lane. Data are representative of at least three independent experiments. (B) The Congo red treatment decreases the *in vivo* activity of PKA. Total protein was extracted from the cells harboring pRS423-pr^{CUP}-*6×MYC-cki1*$^{2-200(S125/130A)}$, and immunoblotting was performed using a mouse anti-Myc antibody. The relative ratio of phosphorylated (Cki1-P) to non-phosphorylated (Cki1) forms of Cki1, normalized against that of untreated cells, is shown below each lane. Data are representative of at least three independent experiments.

Supplementary Figure S8. The lack of PKA-dependent phosphorylation of Msn2 abolishes the effect of *gas1*Δ on rDNA silencing in *gas1*Δ cells. Silencing within the rDNA region was assessed by monitoring the growth of cells (10-fold serial dilutions) plated on SC medium without uracil. SC medium was used as a plating control. The spot assay was performed with WT and *gas1*Δ*msn2*Δ cells containing an empty vector and *gas1*Δ*msn2*Δ cells expressing WT *MSN2* and *msn2*$^{S582D, S620D, S625D, S633D}$.

Supplementary Data

Supplementary Table S1. Yeast strains used in this study

Strain	Genotype	Source
BY4741	*MAT***a** *his3Δ1 leu2Δ0 met15Δ0 ura3Δ0*	Open Biosystems
DMY2798	*MAT***a** *ade2-1 ura3-1 trp1-1 leu2-3,112 his3-11 can1-100 leu2::mURA3*	[6]
DMY2804	*MAT***a** *ade2-1 ura3-1 trp1-1 leu2-3,112 his3-11 can1-100 RDN1-NTS1::mURA3*	[6]
HY0245	*MAT***a** *ade2-1 ura3-1 trp1-1 leu2-3,112 his3-11 can1-100 leu2::mURA3 sir2Δ::TRP1*	This study
HY0291	*MAT***a** *ade2-1 ura3-1 trp1-1 leu2-3,112 his3-11 can1-100 RDN1-NTS1::mURA3 sir2Δ::TRP1*	This study
HY1164	*MAT***a** *ade2-1 ura3-1 trp1-1 leu2-3,112 his3-11 can1-100 leu2::mURA3 gas1Δ::TRP1*	This study
HY1165	*MAT***a** *ade2-1 ura3-1 trp1-1 leu2-3,112 his3-11 can1-100 RDN1-NTS1::mURA3 gas1Δ::TRP1*	This study
HY1167	*MAT***a** *ade2-1 ura3-1 trp1-1 leu2-3,112 his3-11 can1-100 leu2::mURA3 gas1Δ::TRP1 sir2Δ::HIS3*	This study
HY1168	*MAT***a** *ade2-1 ura3-1 trp1-1 leu2-3,112 his3-11 can1-100 RDN1-NTS1::mURA3 gas1Δ::TRP1 sir2Δ::HIS3*	This study
DMY3010	*MAT***a** *ade2-1 ura3-1 trp1-1 leu2-3,112 his3-11 can1-100* $RAD5^+$ *with RDN1::ADE2*	[6]
HY1185	*MAT***a** *ade2-1 ura3-1 trp1-1 leu2-3,112 his3-11 can1-100* $RAD5^+$ *with RDN1::ADE2 gas1Δ::TRP1*	This study
HY0236	*MAT***a** *ade2-1 ura3-1 trp1-1 leu2-3,112 his3-11 can1-100* $RAD5^+$ *with RDN1::ADE2 sir2Δ::TRP1*	This study
HY1448	*MAT***a** *ade2-1 ura3-1 trp1-1 leu2-3,112 his3-11 can1-100* $RAD5^+$ *with RDN1::ADE2 gas1Δ::LEU2 sir2Δ::TRP1*	This study
HY1170	*MAT***a** *his3Δ1 leu2Δ0 met15Δ0 ura3Δ0 MSN2-GFP-HIS3MX6*	This study
HY1171	*MAT***a** *his3Δ1 leu2Δ0 met15Δ0 ura3Δ0 MSN2-GFP-HIS3MX6 gas1Δ::LEU2*	This study

HY1172	*MAT***a** *his3Δ1 leu2Δ0 met15Δ0 ura3Δ0 MSN2-TAP-HIS3MX6*	This study
HY1173	*MAT***a** *his3Δ1 leu2Δ0 met15Δ0 ura3Δ0 MSN2-TAP-HIS3MX6 gas1Δ::URA3*	This study
HY1174	*MAT***a** *his3Δ1 leu2Δ0 met15Δ0 ura3Δ0 PNC1-GFP-HIS3MX6*	This study
HY1175	*MAT***a** *his3Δ1 leu2Δ0 met15Δ0 ura3Δ0 PNC1-GFP-HIS3MX6 gas1Δ::URA3*	This study
HY1176	*MAT***a** *his3Δ1 leu2Δ0 met15Δ0 ura3Δ PNC1-GFP-HIS3MX6 msn2Δ::KanMX4 msn4Δ::LEU2*	This study
HY1177	*MAT***a** *his3Δ1 leu2Δ0 met15Δ0 ura3Δ PNC1-GFP-HIS3MX6 msn2Δ::KanMX4 msn4Δ::LEU2 gas1Δ::URA3*	This study
HY1178	*MAT***a** *his3Δ1 leu2Δ0 met15Δ0 ura3Δ0 SIR2-TAP-HIS3MX6*	This study
HY1179	*MAT***a** *his3Δ1 leu2Δ0 met15Δ0 ura3Δ0 SIR2-TAP-HIS3MX6 gas1Δ::URA3*	This study
HY1180	*MAT***a** *his3Δ1 leu2Δ0 met15Δ0 ura3Δ SIR2-TAP-HIS3MX6 msn2Δ::KanMX4 msn4Δ::LEU2*	This study
HY1181	*MAT***a** *his3Δ1 leu2Δ0 met15Δ0 ura3Δ SIR2-TAP-HIS3MX6 msn2Δ::KanMX4 msn4Δ::LEU2 gas1Δ::URA3*	This study
HY1186	*MAT***a** *ade2-1 ura3-1 trp1-1 leu2-3,112 his3-11 can1-100 RAD5*$^{+}$ *with RDN1::ADE2 msn2Δ::URA3 msn4Δ::LEU2*	This study
HY1187	*MAT***a** *ade2-1 ura3-1 trp1-1 leu2-3,112 his3-11 can1-100 RAD5*$^{+}$ *with RDN1::ADE2 msn2Δ::URA3 msn4Δ::LEU2 gas1Δ::TRP1*	This study
HY1320	*MAT***a** *his3Δ1 leu2Δ0 met15Δ0 ura3Δ0 MSN2-GFP-HIS3MX6* [pRS415]	This study
HY1323	*MAT***a** *his3Δ1 leu2Δ0 met15Δ0 ura3Δ0 MSN2-GFP-HIS3MX6 gas1Δ::URA3* [pRS415]	This study
HY1324	*MAT***a** *his3Δ1 leu2Δ0 met15Δ0 ura3Δ0 MSN2-GFP-HIS3MX6 gas1Δ::URA3* [pRS415 *GAS1-TAP*]	This study
HY1325	*MAT***a** *his3Δ1 leu2Δ0 met15Δ0 ura3Δ0 MSN2-GFP-HIS3MX6 gas1Δ::URA3* [pRS415 *gas1*$^{E161Q,\ E262Q}$*-TAP*]	This study
HY1332	*MAT***a** *his3Δ1 leu2Δ0 met15Δ0 ura3Δ0 MSN2-TAP-HIS3MX6* [pRS415]	This study
HY1335	*MAT***a** *his3Δ1 leu2Δ0 met15Δ0 ura3Δ0 MSN2-TAP-HIS3MX6 gas1Δ::URA3* [pRS415]	This study
HY1336	*MAT***a** *his3Δ1 leu2Δ0 met15Δ0 ura3Δ0 MSN2-TAP-HIS3MX6 gas1Δ::URA3* [pRS415 *GAS1-GFP*]	This study

HY1337	*MAT***a** *his3Δ1 leu2Δ0 met15Δ0 ura3Δ0 MSN2-TAP-HIS3MX6 gas1Δ::URA3* [pRS415 *gas1* $^{E161Q,\ E262Q}$*-GFP*]	This study
HY1402	*MAT***a** *his3Δ1 leu2Δ0 met15Δ0 ura3Δ0 PNC1-TAP-HIS3MX6* [pRS415]	This study
HY1403	*MAT***a** *his3Δ1 leu2Δ0 met15Δ0 ura3Δ0 PNC1-TAP-HIS3MX6 gas1Δ::URA3* [pRS415]	This study
HY1404	*MAT***a** *his3Δ1 leu2Δ0 met15Δ0 ura3Δ0 PNC1-TAP-HIS3MX6 gas1Δ::URA3* [pRS415 *GAS1-GFP*]	This study
HY1405	*MAT***a** *his3Δ1 leu2Δ0 met15Δ0 ura3Δ0 PNC1-TAP-HIS3MX6 gas1Δ::URA3* [pRS415 *gas1* $^{E161Q,\ E262Q}$*-GFP*]	This study
HY1338	*MAT***a** *his3Δ1 leu2Δ0 met15Δ0 ura3Δ0 SIR2-TAP-HIS3MX6* [pRS415]	This study
HY1341	*MAT***a** *his3Δ1 leu2Δ0 met15Δ0 ura3Δ0 SIR2-TAP-HIS3MX6 gas1Δ::URA3* [pRS415]	This study
HY1342	*MAT***a** *his3Δ1 leu2Δ0 met15Δ0 ura3Δ0 SIR2-TAP-HIS3MX6 gas1Δ::URA3* [pRS415 *GAS1-GFP*]	This study
HY1343	*MAT***a** *his3Δ1 leu2Δ0 met15Δ0 ura3Δ0 SIR2-TAP-HIS3MX6 gas1Δ::URA3* [pRS415 *gas1* $^{E161Q,\ E262Q}$*-GFP*]	This study
HY1350	*MAT***a** *ade2-1 ura3-1 trp1-1 leu2-3,112 his3-11 can1-100 leu2::mURA3* [pRS413]	This study
HY1353	*MAT***a** *ade2-1 ura3-1 trp1-1 leu2-3,112 his3-11 can1-100 RDN1-NTS1::mURA3* [pRS413]	This study
HY1356	*MAT***a** *ade2-1 ura3-1 trp1-1 leu2-3,112 his3-11 can1-100 leu2::mURA3 gas1Δ::TRP1* [pRS413]	This study
HY1357	*MAT***a** *ade2-1 ura3-1 trp1-1 leu2-3,112 his3-11 can1-100 leu2::mURA3 gas1Δ::TRP1* [pRS413 *GAS1-GFP*]	This study
HY1358	*MAT***a** *ade2-1 ura3-1 trp1-1 leu2-3,112 his3-11 can1-100 leu2::mURA3 gas1Δ::TRP1* [pRS413 *gas1* $^{E161Q,\ E262Q}$*-GFP*]	This study
HY1359	*MAT***a** *ade2-1 ura3-1 trp1-1 leu2-3,112 his3-11 can1-100 RDN1-NTS1::mURA3 gas1Δ::TRP1* [pRS413]	This study
HY1360	*MAT***a** *ade2-1 ura3-1 trp1-1 leu2-3,112 his3-11 can1-100 RDN1-NTS1::mURA3 gas1Δ::TRP1* [pRS413 *GAS1-GFP*]	This study
HY1361	*MAT***a** *ade2-1 ura3-1 trp1-1 leu2-3,112 his3-11 can1-100 RDN1-NTS1::mURA3 gas1Δ::TRP1* [pRS413 *gas1* $^{E161Q,\ E262Q}$*-GFP*]	This study
HY1344	*MAT***a** *ade2-1 ura3-1 trp1-1 leu2-3,112 his3-11 can1-100 RAD5*$^{+}$ *with RDN1::ADE2* [pRS415]	This study
HY1347	*MAT***a** *ade2-1 ura3-1 trp1-1 leu2-3,112 his3-11 can1-100 RAD5*$^{+}$ *with RDN1::ADE2 gas1Δ::URA3* [pRS415]	This study

HY1348	*MAT***a** *ade2-1 ura3-1 trp1-1 leu2-3,112 his3-11 can1-100 RAD5*$^{+}$ *with RDN1*::*ADE2 gas1*Δ::*URA3* [pRS415 *GAS1-GFP*]	This study
HY1349	*MAT***a** *ade2-1 ura3-1 trp1-1 leu2-3,112 his3-11 can1-100 RAD5*$^{+}$ *with RDN1*::*ADE2 gas1*Δ::*URA3* [pRS415 *gas1*$^{E161Q, E262Q}$*-GFP*]	This study
HY1202	*MAT***a** *his3Δ1 leu2Δ0 met15Δ0 ura3Δ0 TPK1-GFP-HIS3MX6*	This study
HY1203	*MAT***a** *his3Δ1 leu2Δ0 met15Δ0 ura3Δ0 TPK1-GFP-HIS3MX6 gas1*Δ::*URA3*	This study
HY1398	*MAT***a** *his3Δ1 leu2Δ0 met15Δ0 ura3Δ0 TPK1-GFP-HIS3MX6* [pRS415]	This study
HY1399	*MAT***a** *his3Δ1 leu2Δ0 met15Δ0 ura3Δ0 TPK1-GFP-HIS3MX6 gas1*Δ::*URA3* [pRS415]	This study
HY1400	*MAT***a** *his3Δ1 leu2Δ0 met15Δ0 ura3Δ0 TPK1-GFP-HIS3MX6 gas1*Δ::*URA3* [pRS415 *GAS1-TAP*]	This study
HY1401	*MAT***a** *his3Δ1 leu2Δ0 met15Δ0 ura3Δ0 TPK1-GFP-HIS3MX6 gas1*Δ::*URA3* [pRS415 *gas1*$^{E161Q, E262Q}$*-TAP*]	This study
HY1497	*MAT***a** *his3Δ1 leu2Δ0 met15Δ0 ura3Δ0 HIS3MX6*::P_{RPL7B}*-HA-BCY1*	This study
HY1498	*MAT***a** *his3Δ1 leu2Δ0 met15Δ0 ura3Δ0 HIS3MX6*::P_{RPL7B}*-HA-BCY1 gas1*Δ::*URA3*	This study
HY1499	*MAT***a** *his3Δ1 leu2Δ0 met15Δ0 ura3Δ0 HIS3MX6*::P_{RPL7B}*-HA-BCY1 mpk1*Δ::*LEU2*	This study
HY1500	*MAT***a** *his3Δ1 leu2Δ0 met15Δ0 ura3Δ0 HIS3MX6*::P_{RPL7B}*-HA-BCY1 mpk1*Δ::*LEU2 gas1*Δ::*URA3*	This study
HY1390	*MAT***a** *ade2-1 ura3-1 trp1-1 leu2-3,112 his3-11 can1-100 leu2*::*mURA3 gas2*Δ::*TRP1*	This study
HY1391	*MAT***a** *ade2-1 ura3-1 trp1-1 leu2-3,112 his3-11 can1-100 RDN1-NTS1*::*mURA3 gas2*Δ::*TRP1*	This study
HY1392	*MAT***a** *ade2-1 ura3-1 trp1-1 leu2-3,112 his3-11 can1-100 leu2*::*mURA3 gas3*Δ::*TRP1*	This study
HY1393	*MAT***a** *ade2-1 ura3-1 trp1-1 leu2-3,112 his3-11 can1-100 RDN1-NTS1*::*mURA3 gas3*Δ::*TRP1*	This study
HY1394	*MAT***a** *ade2-1 ura3-1 trp1-1 leu2-3,112 his3-11 can1-100 leu2*::*mURA3 gas4*Δ::*TRP1*	This study
HY1395	*MAT***a** *ade2-1 ura3-1 trp1-1 leu2-3,112 his3-11 can1-100 RDN1-NTS1*::*mURA3 gas4*Δ::*TRP1*	This study
HY1396	*MAT***a** *ade2-1 ura3-1 trp1-1 leu2-3,112 his3-11 can1-100 leu2*::*mURA3 gas5*Δ::*TRP1*	This study

HY1397	*MAT***a** *ade2-1 ura3-1 trp1-1 leu2-3,112 his3-11 can1-100 RDN1-NTS1*::*mURA3 gas5*Δ::*TRP1*	This study
HY1388	*MAT***a** *ade2-1 ura3-1 trp1-1 leu2-3,112 his3-11 can1-100 leu2*::*mURA3 bgl2*Δ::*TRP1*	This study
HY1389	*MAT***a** *ade2-1 ura3-1 trp1-1 leu2-3,112 his3-11 can1-100 RDN1-NTS1*::*mURA3 bgl2*Δ::*TRP1*	This study
HY1444	*MAT***a** *ade2-1 ura3-1 trp1-1 leu2-3,112 his3-11 can1-100* $RAD5^+$ *with RDN1*::*ADE2 gas2*Δ::*TRP1*	This study
HY1445	*MAT***a** *ade2-1 ura3-1 trp1-1 leu2-3,112 his3-11 can1-100* $RAD5^+$ *with RDN1*::*ADE2 gas3*Δ::*TRP1*	This study
HY1446	*MAT***a** *ade2-1 ura3-1 trp1-1 leu2-3,112 his3-11 can1-100* $RAD5^+$ *with RDN1*::*ADE2 gas4*Δ::*TRP1*	This study
HY1447	*MAT***a** *ade2-1 ura3-1 trp1-1 leu2-3,112 his3-11 can1-100* $RAD5^+$ *with RDN1*::*ADE2 gas5*Δ::*TRP1*	This study
HY1443	*MAT***a** *ade2-1 ura3-1 trp1-1 leu2-3,112 his3-11 can1-100* $RAD5^+$ *with RDN1*::*ADE2 bgl2*Δ::*TRP1*	This study

Supplementary Table S2. Oligonucleotide primers used for ChIP assays in this study

Locus	Forward Primer	Reverse Primer
rDNA-25S	CGACTAACCCACGTCCAACT	CCGAATGAACTAGCCCTGAA
rDNA-NTS1	TCCCCACTGTTCACTGTTCA	AGGGCTTTCACAAAGCTTCC
rDNA-NTS2/18S	AAGATGCCCACGATGAGACT	GGGAGGTACTTCATGCGAAA
rDNA-18S	CCAGAACGTCTAAGGGCATC	CTCACCAGGTCCAGACACAA
CUP1	TGAAGGTCATGAGTGCCAAT	TTCGTTTCATTTCCCAGAGCA
PNC1 promoter	GATCAAGGTGGCACACAGGG	ATACATAGTGGGCCAAACGG
ACT1	TGACTGACTACTTGATGAAG	ACAGAAGGATGGAACAAAGC

Supplementary Figure S1

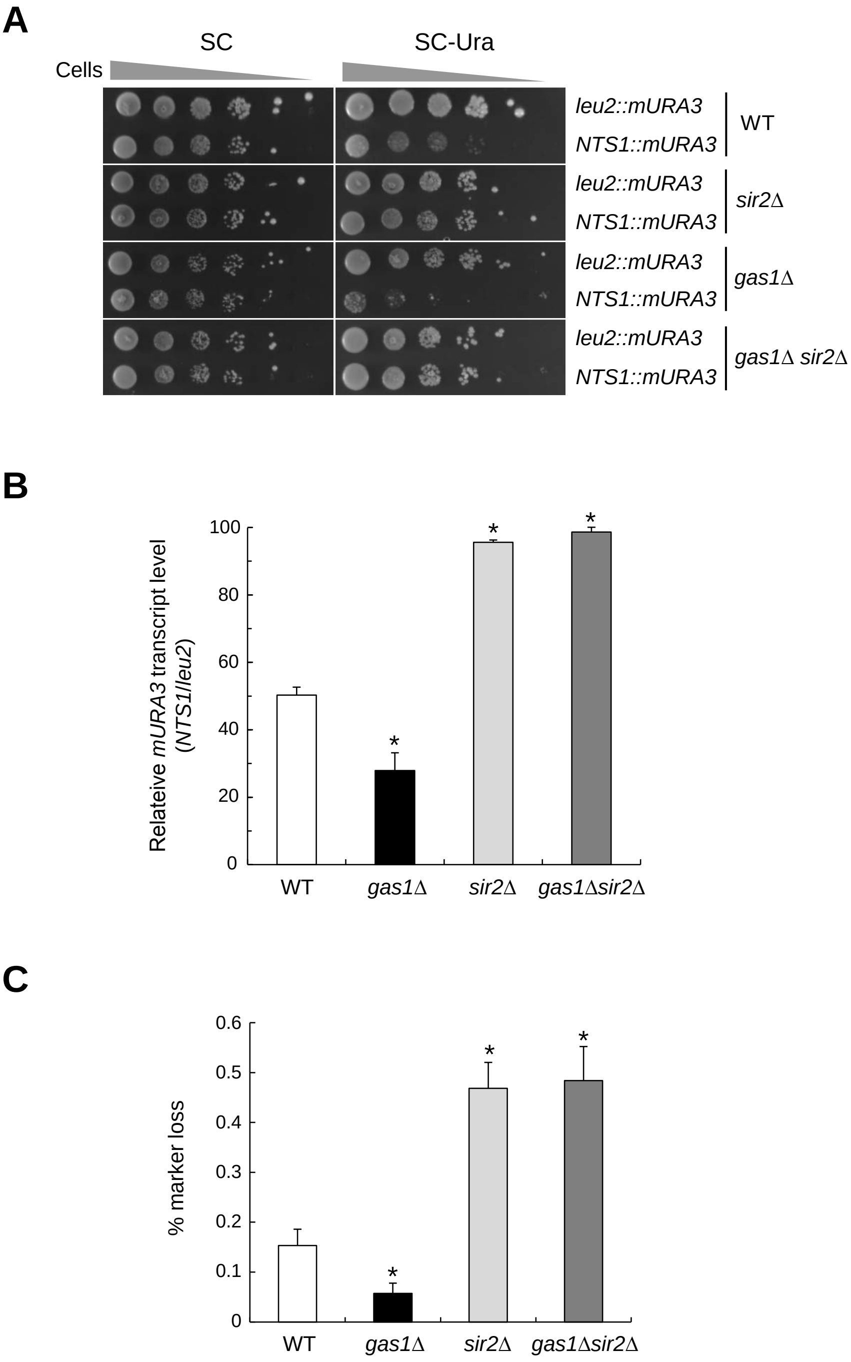

Supplementary Figure S2

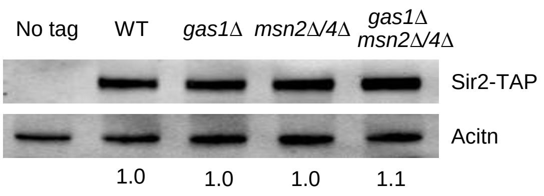

Supplementary Figure S3

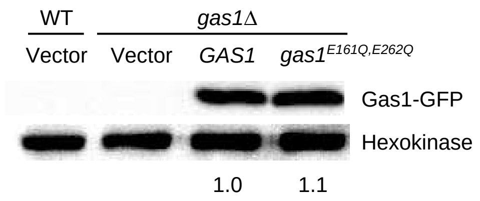

Supplementary Figure S4

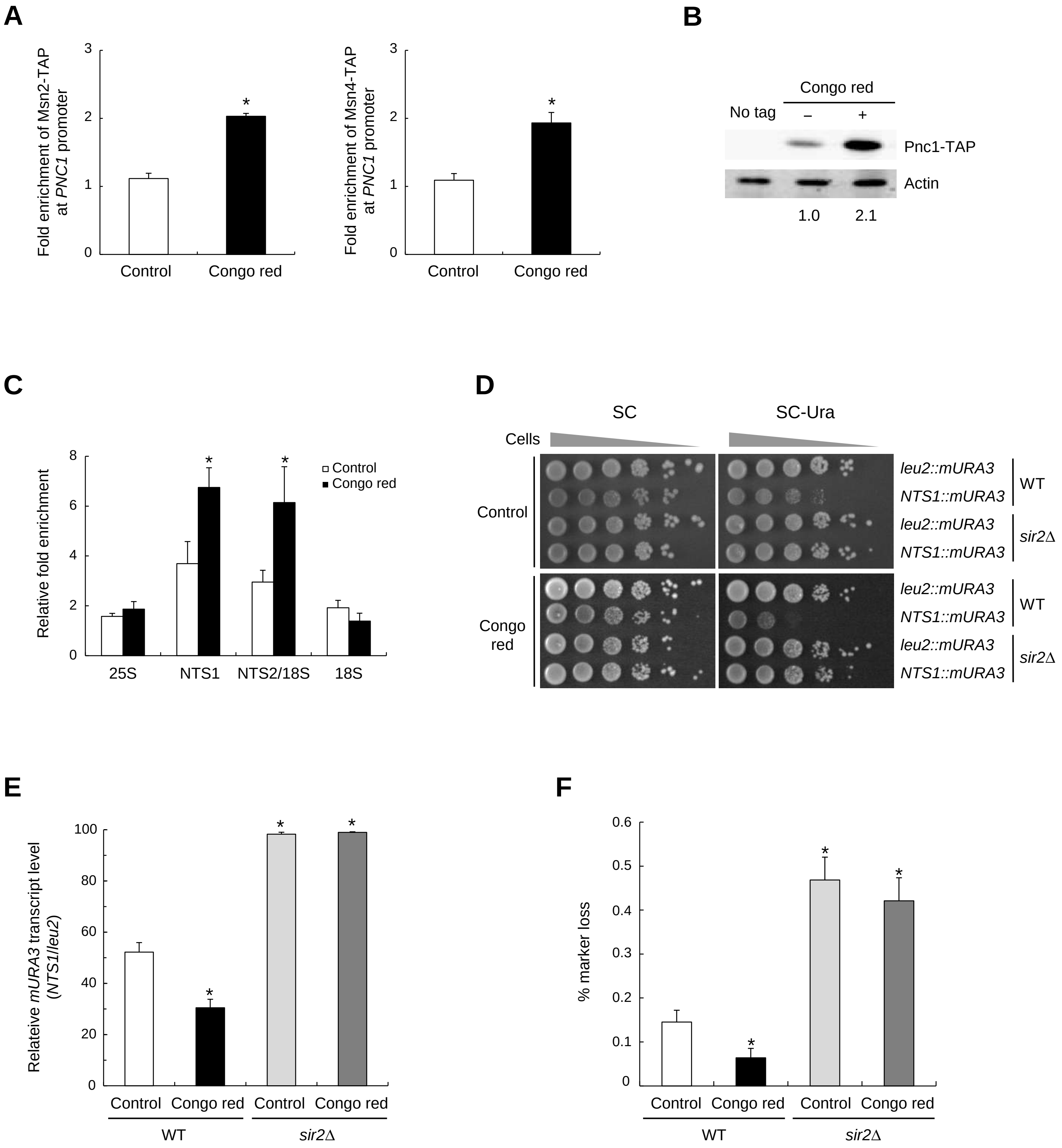

Supplementary Figure S5

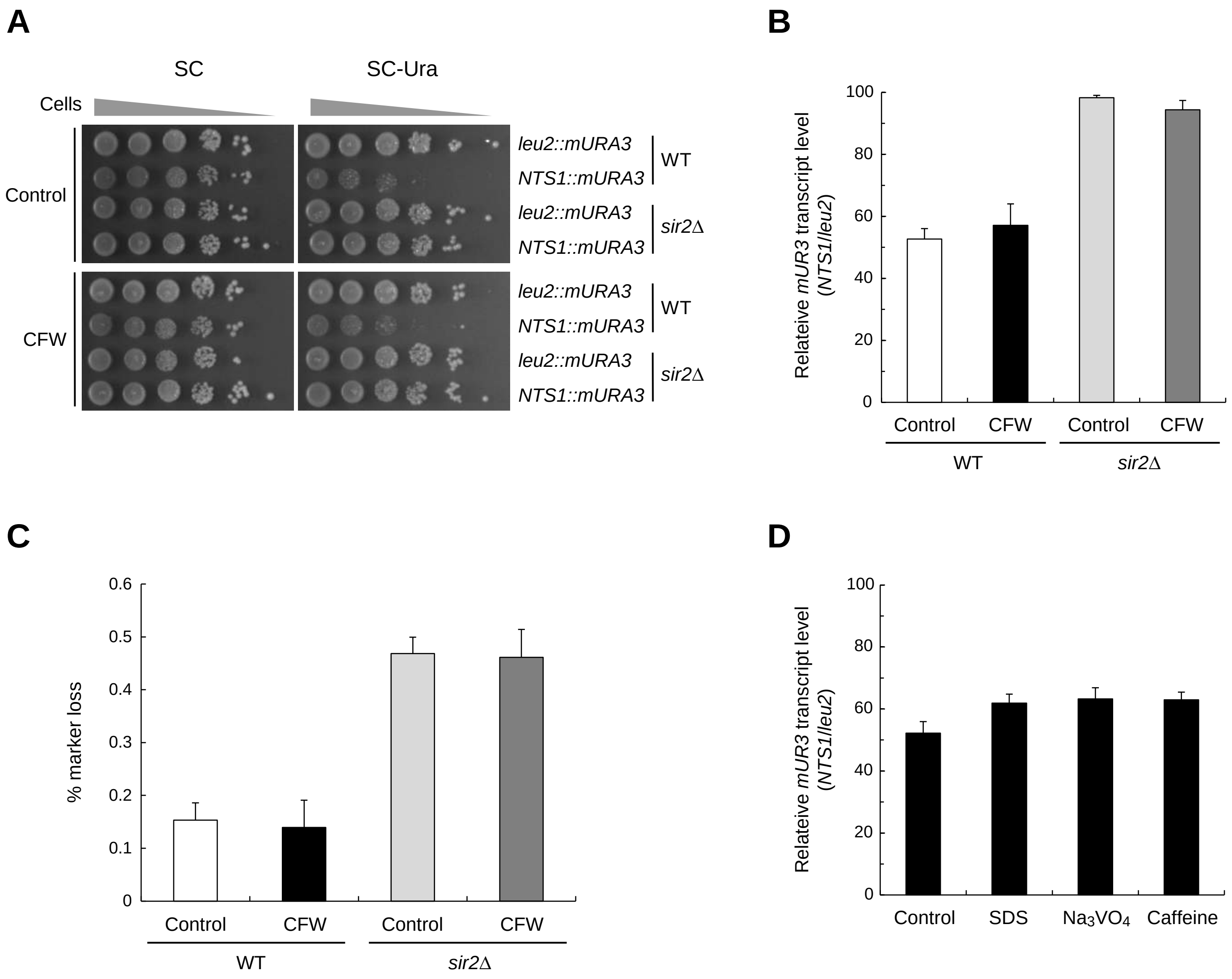

Supplementary Figure S6

A

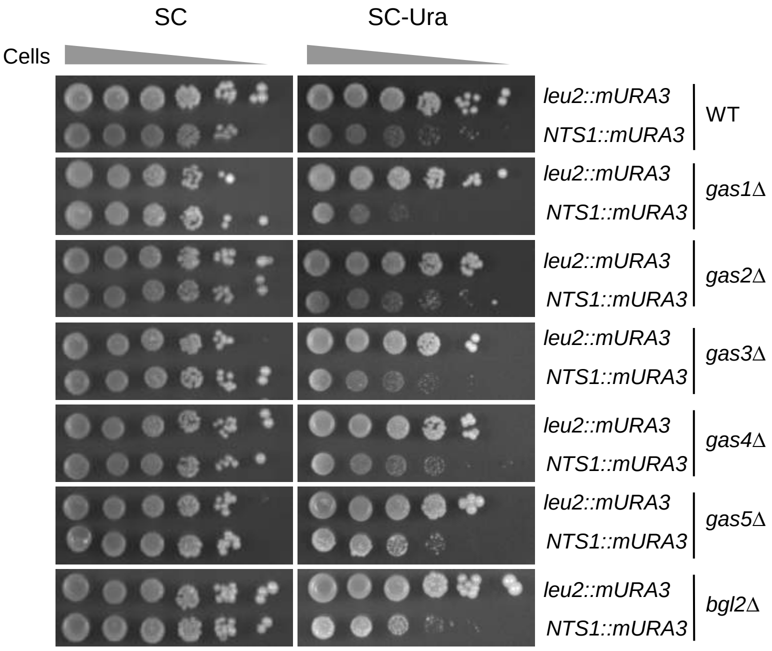

B

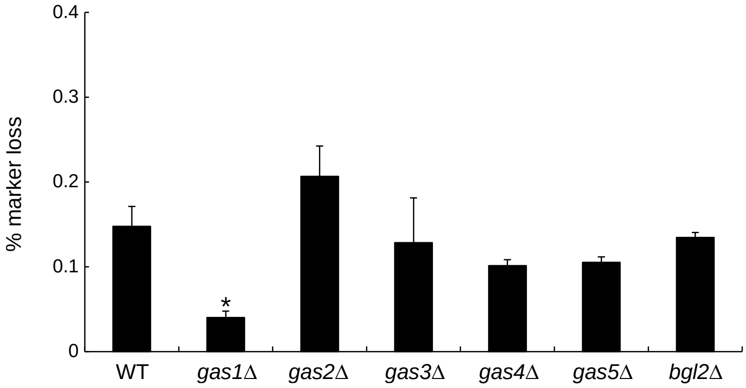

Supplementary Figure S7

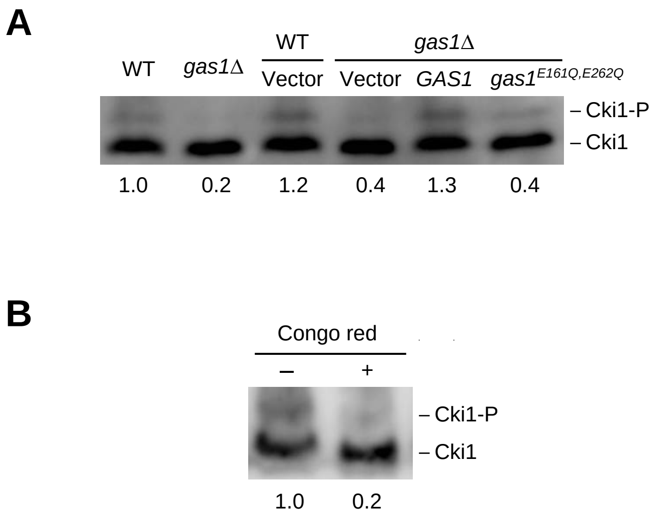

Supplementary Figure S8

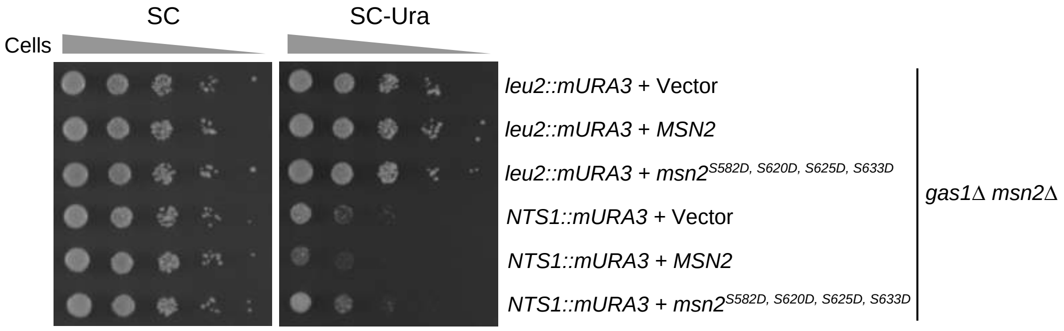

Supplement: SUPPLEMENTARY DATA [file supp_gku570_nar-00010-v-2014-File007.pdf]
